# Non-contact real-time detection of trace nitro-explosives by MOF composites visible-light chemiresistor

**DOI:** 10.1093/nsr/nwac143

**Published:** 2022-07-22

**Authors:** Wei-Hua Deng, Ming-Shui Yao, Min-Yi Zhang, Masahiko Tsujimoto, Kenichi Otake, Bo Wang, Chun-Sen Li, Gang Xu, Susumu Kitagawa

**Affiliations:** State Key Laboratory of Structural Chemistry, Fujian Institute of Research on the Structure of Matter, Chinese Academy of Sciences, Fuzhou 350002, China; University of Chinese Academy of Sciences, Chinese Academy of Sciences, Beijing 100049, China; State Key Laboratory of Structural Chemistry, Fujian Institute of Research on the Structure of Matter, Chinese Academy of Sciences, Fuzhou 350002, China; Institute for Integrated Cell-Material Sciences (WPI-iCeMS), Kyoto University Institute for Advanced Study, Kyoto University, Kyoto 606-8501, Japan; State Key Laboratory of Structural Chemistry, Fujian Institute of Research on the Structure of Matter, Chinese Academy of Sciences, Fuzhou 350002, China; University of Chinese Academy of Sciences, Chinese Academy of Sciences, Beijing 100049, China; Institute for Integrated Cell-Material Sciences (WPI-iCeMS), Kyoto University Institute for Advanced Study, Kyoto University, Kyoto 606-8501, Japan; Institute for Integrated Cell-Material Sciences (WPI-iCeMS), Kyoto University Institute for Advanced Study, Kyoto University, Kyoto 606-8501, Japan; Beijing Key Laboratory of Photoelectronic/Electrophotonic Conversion Materials, Key Laboratory of Cluster Science, Ministry of Education, School of Chemistry and Chemical Engineering, Beijing Institute of Technology, Beijing 100081, China; State Key Laboratory of Structural Chemistry, Fujian Institute of Research on the Structure of Matter, Chinese Academy of Sciences, Fuzhou 350002, China; University of Chinese Academy of Sciences, Chinese Academy of Sciences, Beijing 100049, China; State Key Laboratory of Structural Chemistry, Fujian Institute of Research on the Structure of Matter, Chinese Academy of Sciences, Fuzhou 350002, China; University of Chinese Academy of Sciences, Chinese Academy of Sciences, Beijing 100049, China; Fujian Science & Technology Innovation Laboratory for Optoelectronic Information of China, Fuzhou 350108, China; Institute for Integrated Cell-Material Sciences (WPI-iCeMS), Kyoto University Institute for Advanced Study, Kyoto University, Kyoto 606-8501, Japan

**Keywords:** MOFs, metal oxides, electrical devices, thin films, gas sensors

## Abstract

To create an artificial structure to remarkably surpass the sensitivity, selectivity and speed of the olfaction system of animals is still a daunting challenge. Herein, we propose a core-sheath pillar (CSP) architecture with a perfect synergistic interface that effectively integrates the advantages of metal–organic frameworks and metal oxides to tackle the above-mentioned challenge. The sheath material, NH_2_-MIL-125, can concentrate target analyte, nitro-explosives, by 10^12^ times from its vapour. The perfect band-matched synergistic interface enables the TiO_2_ core to effectively harvest and utilize visible light. At room temperature and under visible light, CSP (TiO_2_, NH_2_-MIL-125) shows an unexpected self-promoting analyte-sensing behaviour. Its experimentally reached limit of detection (∼0.8 ppq, hexogeon) is 10^3^ times lower than the lowest one achieved by a sniffer dog or all sensing techniques without analyte pre-concentration. Moreover, the sensor exhibits excellent selectivity against commonly existing interferences, with a short response time of 0.14 min.

## Teaser

A core-sheath pillararray constructed by metal-organic frameworks sheath and metal oxide core with a unique photo-activated sensing interfaceshows unprecedented nitro-explosivedetection performances far beyond a sniffer dog.

## INTRODUCTION

Olfaction is a sense of gaseous matter (smell or odours) associated with signs of attraction, safety, danger, etc. Olfaction occurs when odorant molecules chemically bind to specific sites on receptors located in the nasal cavity, integrating with other organs to form the sense of odour [1]. At present, the detection limit (LOD) of well-trained sniffer dogs is at around the hundreds of parts per trillion (ppt) range [2,3]. To mimic the olfaction of animals, non-contact and real-time detection techniques based on chemical interaction are required and related sensing materials have been intensively studied. Although significant progress has been achieved, the sensitivity of the best chemical gas-sensing technique is similar to those of animals and at the ppt level [4–7]. The question is still open on whether it is possible to create an artificial system to remarkably surpass the sensitivity, selectivity and speed of the olfaction system of animals.

**Figure 1.**
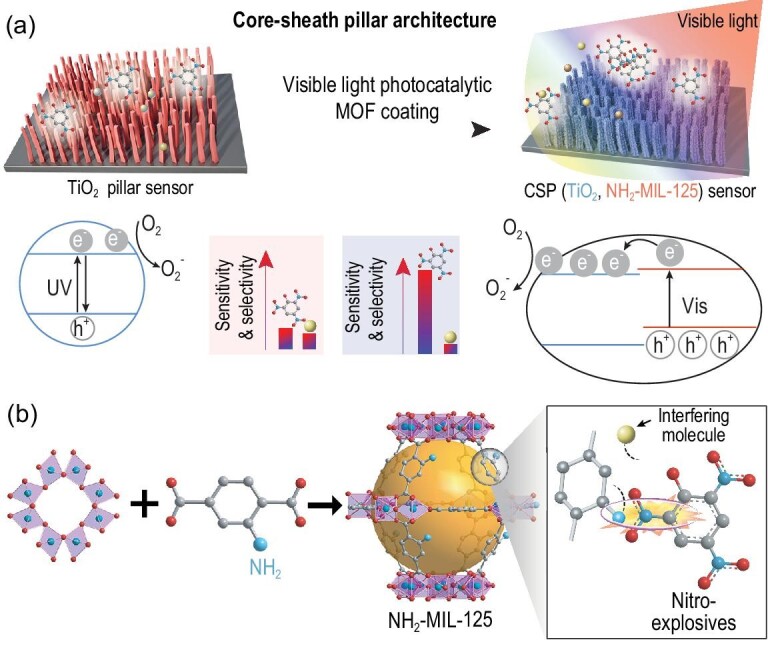
Design of the CSP architecture and the specific interaction. (a) Design of visible-light-activated CSP (TiO_2_, NH_2_-MIL-125) chemiresistive material for nitro-explosive detection at RT. (b) Schematic of the structure of NH_2_-MIL-125 and the specific interaction of its organic ligand with nitro-explosives (golden ball: the cavity of NH_2_-MIL-125).

Metal oxides (MOs) nanostructure-based chemiresistive gas-sensing techniques are among the most promising candidates to positively answer the above question [8,9]. However, due to the low specific area and broad-spectrum responses, metal-oxide sensing materials still cannot surpass the olfaction system of animals [10–14]. To overcome these problems, we designed a combined material containing target-selective and reactive materials for the detection of a part-per-quadrillion (ppq, = 10^–3^ ppt)-level molecule, with a corn-dog-like core-sheath pillar (CSP) architecture (Fig. 1a). Accordingly, an emerging class of crystalline microporous materials, metal–organic frameworks (MOFs) or porous coordination polymers (PCPs) [15–24], designed with a high affinity to analytes, are coated on the MO to adsorb selectively and locally concentrate target molecules [25,26], while the MO provides the active sites for the sensing reaction and conducts the electrical sensing signal. The essential question of CSP (MO, MOF) is how to create a perfect energy-band-matched MOF/MO interface that can effectively generate and separate light-excited charge carriers to produce active oxygen species. MOFs can couple with MOs to achieve the desired visible-light-active and analyte-accessible MOF/MO interface [27].

In this work, nitro-explosives that possess ultra-low saturated vapour pressures at RT, e.g. 0.97 ppb of 2,4,6-trinitrophenol (TNP), 9.1 ppb of 2,4,6-trinitrotoluene (TNT) and 4.9 ppt (Robert G. Ewing *et al*. recently reported 30 ± 10 ppt) of hexogen (RDX) were selected as the analytes to reveal the potential of CSP (MO, MOF) [25,28–30]. A highly stable MOF, NH_2_-MIL-125, was selected as a sheath material based on the following considerations (Fig. 1b): (i) it not only possesses local concentration ability but can also form a visible-light-active interface with TiO_2_, which can be used to enhance the sensitivity towards nitro-explosives; (ii) its amino groups interact strongly with nitro-explosives, which is favourable for the selectivity towards the latter. As a proof of concept, a layer of 15-nm-thick NH_2_-MIL-125 thin film was epitaxially grown on TiO_2_ pillars to form a CSP (TiO_2_, NH_2_-MIL-125) visible-light-chemiresistive sensing material for RT nitro-explosive detection. Consequently, CSP (TiO_2_, NH_2_-MIL-125) showed an experimentally detected concentration as low as 0.8 ppq of RDX vapour [30]. Notably, this value is 10^3^ times lower than the lowest experimental LOD for all gaseous molecules achieved by all sensing techniques without analyte pre-concentration [2,4]. CSP (TiO_2_, NH_2_-MIL-125) achieved non-contact and real-time detection of RDX with an amount as low as 5 mg and a distance as long as 8 m, which is also comparable with the gold standard [2–4]. Moreover, it also showed excellent selectivity in discriminating various nitro-explosives, such as RDX, TNT and TNP, among 25 structurally similar or commonly existing interferences.

## RESULTS AND DISCUSSION

**Figure 2.**
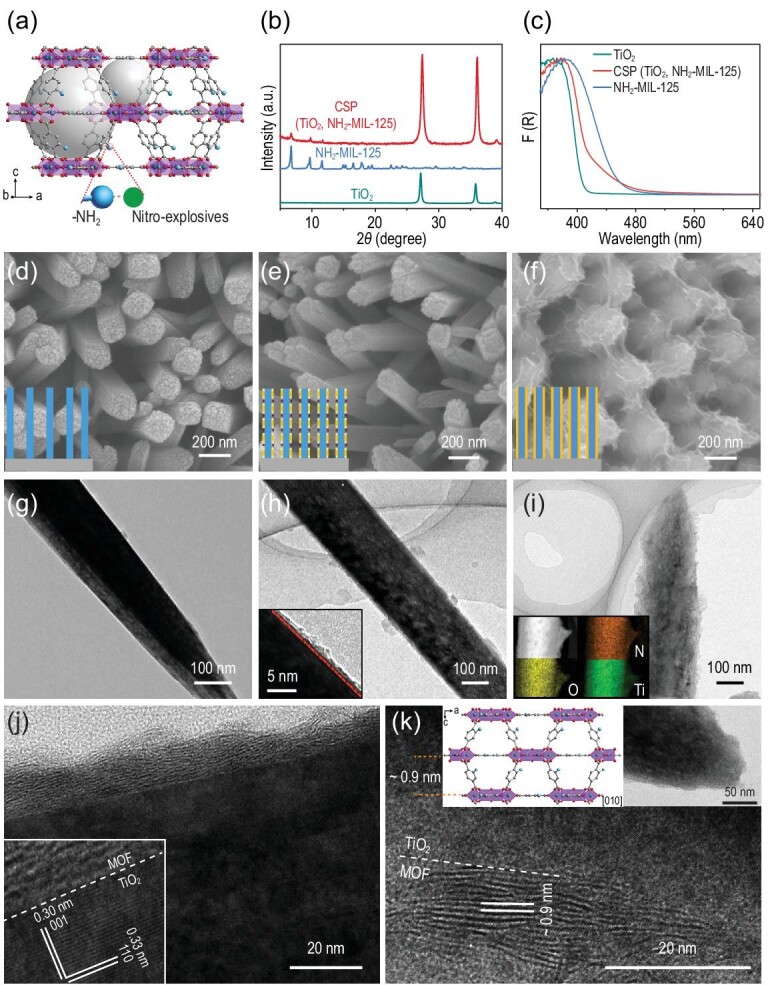
Structural characterizations of the CSP architecture. (a) Perspective view of NH_2_-MIL-125; the gray balls indicate the centres of the octahedral and tetrahedral vacancies in NH_2_-MIL-125. (b) PXRD patterns of TiO_2_, NH_2_-MIL-125 and CSP (TiO_2_, NH_2_-MIL-125); (c) DRS spectra of TiO_2_, NH_2_-MIL-125 and CSP (TiO_2_, NH_2_-MIL-125). SEM and TEM images of (d and g) vertically aligned TiO_2_ pillars, (e and h) TiO_2_ pillars after two-step seeding, and (f, i–k) CSP (TiO_2_, NH_2_-MIL-125) (insets in (d–f) are schematics of the growth of CSP; insets in (i) are N, O and Ti EDX mapping images of the side view of a CSP).

In the crystal structure of NH_2_-MIL-125, a large number of amino groups are attached to the skeleton of NH_2_-MIL-125 (Fig. 2a), which are not coordinated with other inorganic components, and are thus selectively interacting with nitro-explosives through the windows in the cages in our designed product [31,32]. The reported specific surface area of NH_2_-MIL-125 reaches 1300 m^2^ g^−1^, which is sufficient to locally concentrate nitro-explosives [33]. Although various composite materials of TiO_2_ and NH_2_-MIL-125 have been reported previously [34,35], it is the very first time a unique CSP architecture of TiO_2_@NH_2_-MIL-125 (CSP (TiO_2_, NH_2_-MIL-125)) has been introduced, where the MOF sheath, constructed from intergrown ultra-thin nanosheets, was quasi-oriented and uniformly coated on TiO_2_. A two-step seed-assisted solvothermal method was developed to fabricate this type of CSP architecture (see ‘Methods’ section for details). To be more specific, the vertically oriented TiO_2_ pillars were first grown on an Al_2_O_3_ substrate (inset in Fig. 2d). The TiO_2_ posts were then immersed in solutions of BDC-NH_2_ (2-aminobenzenedicarboxylate) ligand and heated. After washing, the TiO_2_ pillars were immersed in titanium n-butoxide solution and heated to grow NH_2_-MIL-125 seeds (inset in Fig. 2e). The NH_2_-MIL-125 seed-modified TiO_2_ pillars were then placed in a Teflon-lined autoclave containing a solution of BDC-NH_2_ and titanium n-butoxide. After being maintained at 150°C for 3 days, CSP (TiO_2_, NH_2_-MIL-125) was obtained (inset in Fig. 2f).

Powder X-ray diffraction (PXRD) measurements revealed peaks at 2*θ* < 25° belonging to NH_2_-MIL-125 (Fig. 2b) [31,33]. UV–vis diffuse reflectance spectroscopy (UV–vis DRS) of TiO_2_ and CSP (TiO_2_, NH_2_-MIL-125) clearly showed that the NH_2_-MIL-125 sheath is a good visible-light sensitizer, as it significantly increases the absorption cross section of TiO_2_ from 410 to 530 nm, which improves its light-harvesting efficiency (Fig. 2c). Scanning electron microscopy (SEM) and transmission electron microscopy (TEM) images of TiO_2_ pillars and CSP (TiO_2_, NH_2_-MIL-125) on Al_2_O_3_ substrates are shown in Fig. 2d–i. TiO_2_ pillars are vertically aligned with well-defined surfaces (Fig. 2d and g). The average diameter and length of the TiO_2_ pillars are ∼150 nm and ∼1.5 μm, respectively. After seeding, crystalline nuclei of the MOF material were formed (Fig. 2e and h). CSP (TiO_2_, NH_2_-MIL-125) (Fig. 2f and i) has a core-sheath structure, where a uniform MOF thin film sheath is constructed from intergrown ultra-thin nanosheets, which is confirmed by the uniformly distributed N, O and Ti on the MOF sheath shown in energy-dispersive X-ray spectroscopy (EDX) maps (insets in Fig. 2i). The thickness of the MOF layer is ∼15 nm. The homogeneous coating of NH_2_-MIL-125 on the TiO_2_ pillar is further confirmed by high-magnification TEM images of its side face (Fig. 2j) and tip face (Fig. 2k), from which the lattice spacing of both TiO_2_ and NH_2_-MIL-125, as well as their clean interfaces, can be observed. Interestingly, the observed similar lattice spacing of ∼0.9 nm of the MOF sheath also indicates their quasi-oriented growth along one direction on both the side and top surfaces of the TiO_2_ pillar, which might be beneficial for charge transfer between them. Considering the stacking differences of inorganic Ti–O nodes along the [010] and [001] directions (insets of Fig. 2k), the oriented plane could be indexed as (002). The lattice fringes (inset in Fig. 2j) and the aligned dots in the selected-area electron diffraction pattern (Supplementary Fig. S1) confirm that the TiO_2_ pillars have a single crystal rutile phase and grow along the [001] direction.

**Figure 3.**
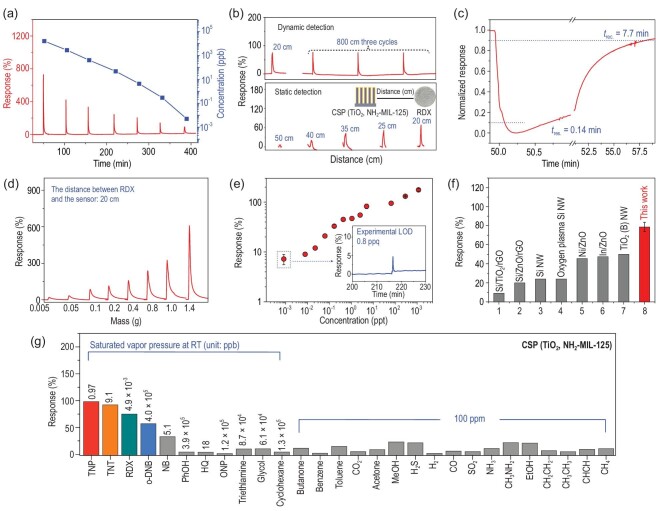
Sensing properties of CSP (TiO_2_, NH_2_-MIL-125) toward RDX under visible light and at RT. (a) Dynamic response–recovery curve for RDX at different expected concentrations under visible light (the distance between RDX and the sensor: 20 cm); (b) static and dynamic response–recovery curves for different distances from 1.4 g of RDX (vapour pre-concentrated at RT); (c) normalized response–recovery curve to 15.7 ppm of RDX vapour (expected concentration); (d) dynamic response–recovery curve with different masses (vapour pre-concentrated at 150^o^C); (e) log–log plots of response–concentration with experimental LOD values (the inset is the response toward 0.8 ppq RDX vapour pre-concentrated at 253K); (f) response comparisons of different gas-sensing materials toward saturated RDX vapours at RT [11–14,28,37,38]; (g) response comparisons among nitro-explosives and interference vapours.

The real-time detection of RDX by CSP (TiO_2_, NH_2_-MIL-125) was conducted under simulated static-state and dynamic-state conditions in a homemade characterization system reported in our previous work (see Supplementary information and Supplementary Fig. S2 for details) [36]. Silver paste was coated on both ends of the film of CSP (TiO_2_, NH_2_-MIL-125) as electrodes and then these were placed inside a sealed quartz chamber with dry air as the cleaning and carrier gas. Without light irradiation, CSP (TiO_2_, NH_2_-MIL-125) showed nearly no sensing response to RDX vapour (Supplementary Fig. S3). Conversely, under visible-light (420–790 nm) irradiation, CSP (TiO_2_, NH_2_-MIL-125) showed a distinct response to RDX in both static and dynamic tests (Fig. 3a and Supplementary Fig. S4).

Although the static test is unsuitable for practical application in real-time detection, its result represents the thermodynamic maximum at RT (Supplementary Fig. S4). This response gives a value reaching 82%, which is ∼164% higher than the highest value reported for chemiresistive sensors (Fig. 3f) [11–14,28,37,38]. Concentration and mass-dependent dynamic tests can mimic the vapour-diffusion-induced concentration gradient or dynamic explosive residues in practical applications. CSP (TiO_2_, NH_2_-MIL-125) shows a dynamic response and recovery to the RDX vapour with concentrations ranging from 4.9 ppt to 15.7 ppm at high signal-to-noise ratios (Fig. 3a). Its response increases with the increment of concentration of RDX vapour and its high sensitivity results in a response of ca. 730% when detecting 15.7 ppm RDX. Good repeatability of the response to 4.9 ppt and 15.7 ppm RDX with a coefficient of variation as low as 0.8% and 7.3%, respectively, for five successive cycles was also observed (Supplementary Fig. S5). Real-time response–recovery curves of three different CSP (TiO_2_, NH_2_-MIL-125) sensors fabricated in different batches upon exposure toward 15.7 ppm RDX under visible light showed responses of 730%, 742% and 726%, respectively. The CV (coefficient of variation) is only 1.1%, which showed good reproducibility (Supplementary Fig. S6).

The response to 4.9 ppt RDX was calculated as 75%, which is close to the value obtained in the static test and 258% higher than those of TiO_2_ (Supplementary Figs S4 and S19b, and Fig. 3g). CSP (TiO_2_, NH_2_-MIL-125) also showed a mass-dependent response to RDX powder (Fig. 3d), where a response of 20% to 5 mg RDX and 610% to 1.4 g RDX was observed. The lowest detectable concentration of RDX vapour was obtained by controlling the evaporation temperature of RDX at 253 K (Fig. 3e, Supplementary Fig. S7 and Supplementary Table S1) [29,30]. A noticeable response to 0.8 ppq was observed (insets in Fig. 2e). Moreover, the response curve possesses a high signal-to-noise level, which is robust evidence to demonstrate that the LOD to RDX can be <0.8 ppq in theory. The LOD of CSP (TiO_2_, NH_2_-MIL-125) toward TNT and TNP vapour was also deduced to be as low as 0.696 and 1.92 ppt, respectively, by setting the response as 10% in the log–log plots of response vs concentration (Supplementary Figs S8 and S9, and Supplementary Tables S2 and S3). The response and recovery time were estimated to be 0.14 and 7.7 min (Fig. 3c), respectively. This is a very fast response and is sufficient for real-time detection. These results allow the real-time detection of trace nitro-explosive vapours.

The influence of the light source on the response was also investigated. CSP (TiO_2_, NH_2_-MIL-125) has an optimized performance at a power density of 320 mW cm^–2^ and a wavelength of 475 nm (Supplementary Figs S10–S12 and Supplementary Table S4). Moreover, CSP (TiO_2_, NH_2_-MIL-125) shows good long-term baseline stability under ambient or high humidity circumstances (100% RH) for >5 months (Supplementary Fig. S13).

The high sensitivity of CSP (TiO_2_, NH_2_-MIL-125) allows it to detect nitro-explosives without physical contact with solid samples. As an example to mimic real-world sensing, the saturated vapour generated from 1.4 g RDX powder was allowed to freely diffuse in a quartz chamber without carrier gas to form a concentration gradient (static detection). The responses of CSP (TiO_2_, NH_2_-MIL-125) at different distances from RDX powder were recorded and are shown in Fig. 3b. The results clearly revealed the ability of CSP (TiO_2_, NH_2_-MIL-125) to discriminate gram levels of RDX from distances reaching 50 cm. In a more practical condition with a flowing air of 60 sccm (dynamic detection), CSP (TiO_2_, NH_2_-MIL-125) showed remarkably extended detectable distance and a response value at 8 m is similar to that at 20 cm. Since more RDX can produce the same concentration of vapour over a larger range, CSP (TiO_2_, NH_2_-MIL-125) is expected to detect kilogram levels of RDX at much longer distances.

Compared with TiO_2_, CSP (TiO_2_, NH_2_-MIL-125) possesses significantly enhanced selectivity (Fig. 3g). It shows a good response to typical nitro-explosives, such as TNP, TNT and RDX (Fig. 3 and Supplementary Fig. S14), while it shows a poor response to 25 other interfering gas molecules including phenol (PhOH), hydroquinol (HQ), o-nitrophenol (ONP), benzene, toluene, CO_2_, acetone, methanol, H_2_S, etc. (Fig. 3 and Supplementary Figs S15–S21). This unique selectivity permits the discrimination of these nitro-explosives from structurally similar or commonly existing interfering gas molecules. H_2_O is one of the most common interference vapours. However, this problem can be solved well by simply filtering the analyte vapour with three layers of the hydrophobic cloth obtained from disposable medical masks. As shown in Supplementary Fig. S22a, the hydrophobic mask clothes would suppress the response of humidity. As a result, >90% of the original value (730%, dry air as carrier gas) toward the 15.7 ppm of RDX vapour is maintained even under 95% RH (660%, 95% RH air as carrier gas) (Supplementary Fig. S22b).

**Figure 4.**
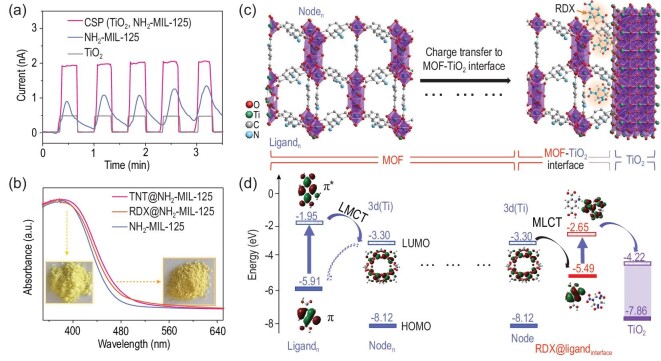
Sensing mechanisms of CSP (TiO_2_, NH_2_-MIL-125) toward RDX under visible light and at RT. (a) Photocurrent of CSP (TiO_2_, NH_2_-MIL-125), NH_2_-MIL-125 and TiO_2_ under visible light; (b) absorption of NH_2_-MIL-125 after exposure to RDX vapour; (c) structures of TiO_2_, NH_2_-MIL-125 and their interface exposed to RDX vapour (the orange molecules) and (d) energy-level diagram of ligand, metal node and RDX@ligand relative to TiO_2_, and frontier molecular orbitals of ligand, metal node and RDX@ligand showing thermodynamically favoured charge-carrier separations and transfer, as well as self-promoting analyte-sensing behaviour.

Compared with TiO_2_ and NH_2_-MIL-125, CSP (TiO_2_, NH_2_-MIL-125) showed much higher sensitivity in detecting nitro-explosives, which may be ascribed to the following three reasons: (i) NH_2_-MIL-125 possesses a high specific surface area to locally concentrate nitro-explosives; (ii) NH_2_-MIL-125 significantly increases the absorption cross section of TiO_2_ from 410 to 530 nm to improve its light-harvesting efficiency, thus enhancing its photocurrent (Fig. 4a); (iii) with the MOF sheath, our density functional theory (DFT) calculations (Fig. 4c and d, and Supplementary Fig. S23) reveal thermodynamically favoured charge-carrier separation and transfer at the staggered-gap (type II) heterojunction of the MOF–TiO_2_ interface. Thus, the MOF–TiO_2_ heterojunction can act as a ‘pump’ to extract the photo-excited electrons generated at both the MOF sheath and MOF–TiO_2_ interface to enable TiO_2_ to produce active oxygen species (O_2_^–^) for the sensing reaction [27]. In this case, RDX will undergo charge-transfer reactions with the O_2_^–^ ions (Supplementary Equations S1 and S2), thus NO_2_*^–^* ions and perhaps other ions (explosive negative ions, NO_2_, NH_3_, CO_2_, etc.) are formed. More interestingly, at the MOF–TiO_2_ interface, when nitro-explosives (TNP, TNT, RDX, o-DNB) bind to the ligand, a new band structure is formed, which results in an unexpected self-promoting analyte-sensing behaviour (Fig. 4 and Supplementary Figs S23–S26) [39,40]. Theoretical calculation revealed that this new band structure of nitro-explosives@BDC-NH_2_ would: (i) result in a much smaller energy gap than that of ligand and common interferences@ligand (calculated common interferences: PhOH, acetone, toluene, benzene, Supplementary Table S5), and would extend the light-absorption range (Fig. 4b); (ii) more importantly, induce the photo-excited electrons transfer from the highest occupied molecular orbital (HOMO) mainly located on the ligand part to the lowest unoccupied molecular orbital (LUMO) mainly located on the nitro-explosives part, which promotes the separation and transfer of the photo-excited charge carriers from the MOF to the interface (Supplementary Table S6).

After being absorbed by the MOF sheath, TNT showed a smaller HOMO–LUMO gap (Supplementary Table S5 and Supplementary Fig. S25) and higher calculated intermolecular electronic coupling (*J*_eff_) (Supplementary Table S6) than RDX. Meanwhile, TNT has three orders of magnitude higher saturated vapour pressure than RDX at room temperature. Thus, CSP (TiO_2_, NH_2_-MIL-125) should have a much higher response difference between TNT and RDX than the current results. This abnormal phenomenon can be attributed to the pre-concentration effects of MOF sheath on nitro-explosive vapours, which showed the superior pre-concentration efficiency of RDX over TNT, as experimentally confirmed by the mass-transduced adsorption results of a commercial microcantilever (Supplementary Figs S27–S29). Specifically, NH_2_-MIL-125 was astonishingly found to concentrate the vapour of solid nitro-explosive, TNT and RDX, by 10^9^ and 10^12^ times, respectively. The estimated density of TNT and RDX in NH_2_-MIL-125 was very high, reaching 3.56 × 10^–2^ and 3.15 × 10^–2^ g cm^–3^, respectively, which are close to those of solid TNT (1.6 g cm^–3^) and RDX (1.9 g cm^–3^). It significantly reduces the concentration differences of RDX and TNT at the interface of MOF and TiO_2_, thereby resulting in response values with less difference.

Moreover, it is well known that apart from acid–base pairing interactions, electron-rich -NH_2_ groups form strong charge-transfer complexing interactions with electron-deficient nitro-explosives [40]. This interaction was confirmed by the colour of the NH_2_-MIL-125 changing from yellow to brown upon exposure to the RDX or TNT vapour (Fig. 4b). The calculated charge density difference plots confirmed the above experimental results and showed the charge depletion of the -NH_2_ group of the ligand and the obvious charge increase of the -NO_2_ groups of nitro-explosives on their interacted surfaces (Supplementary Fig. S26). The formation of the charge-transfer complexes between NH_2_-MIL-125 and nitro-explosives may result in the high selectivity of CSP (TiO_2_, NH_2_-MIL-125) to nitro-explosives.

## CONCLUSION

Aiming to create an artificial olfaction system that surpasses the sensitivity, selectivity and sensing speed of the olfaction system of a sniffer dog, we, for the very first time, introduced a CSP (TiO_2_, NH_2_–MIL–125) chemiresistive sensing material to fulfil the urgent but unsatisfied requirements with respect to the non-contact and real-time detection of nitro-explosives. It showed excellent sensitivity and selectivity: (i) this material achieved the highest response (82%) to saturated RDX vapour (4.9 ppt) and the lowest detectable concentration (∼0.8 ppq) among all the reported chemiresistive materials and realized the detection of an amount of RDX as low as 5 mg and a distance of ≤8 m in a non-contact, real-time manner; (ii) it could discriminate nitro-explosives, such as TNP, TNT and RDX, among 25 structurally similar or commonly existing interferences. Since the crystal structure of the MOF sheath can be flexibly designed to realize the required properties, the CSP (MO, MOF) architecture not only offers much room for further optimization for nitro-explosive detection, but might also provide a general method for developing high-performance sensing materials for detecting other crucial chemicals, such as various volatile organic compounds, such as NO_x_ and SO_x_.

## METHODS

### Preparation of TiO_2_ pillars

The Al_2_O_3_ substrates were cut into 8 × 10 mm^2^ square pieces and thoroughly cleaned by ultrasonication in a mixture solution (water:isopropanol:acetone = 1:1:1), followed by drying with N_2_ gas. Rutile TiO_2_ NWAs were grown on an Al_2_O_3_ substrate with a hydrothermal method. To facilitate the growth of NWAs, a layer of TiO_2_ seed was first deposited on the Al_2_O_3_ substrate by thermally decomposing titanium n-butoxide (Ti(OC_4_H_9_)_4_, TNB) at 450°C. Meanwhile, 0.4 mL of titanium n-butoxide was mixed with 12 mL of 6 M HCl in a 20-mL Teflon-lined autoclave. The seeded substrate was then up-down immersed into the solution. The hydrothermal reaction was conducted in an electric oven at 150°C for 4 h and then slowly cooled down to room temperature. Subsequently, the TiO_2_ NWAs covered in Al_2_O_3_ were rinsed thoroughly with deionized (DI) water and dried in air. The as-synthesized TiO_2_ NWAs were further annealed in air at 450°C for 30 min to improve their crystallinity and conductivity.

### Preparation of CSP (TiO_2_, NH_2_-MIL-125)

A two-step seed-assisted solvothermal method was developed to grow a NH_2_-MIL-125 sheath on the surface of TiO_2_ pillars. First, TiO_2_ pillars were face-down immersed into a mixed solution containing 2-aminoterephthalate (BDC-NH_2_), dimethyl formamide (DMF) and methanol in a Teflon-lined autoclave. The sealed autoclave was kept at 150°C for 12 h. TiO_2_ pillars were immersed into a mixed solution of TNB, DMF and methanol in a Teflon-lined autoclave. The sealed autoclave was heated to 150°C and kept for 4 h, before cooling down to RT. Finally, the MOF seeded TiO_2_ pillars were obtained. The seeded sample was up-down immersed into a mixed solution containing BDC-NH_2_, TNB, DMF and methanol in a Teflon-lined autoclave and heated at 150°C for 72 h. After that, CSP (TiO_2_, NH_2_-MIL-125) was obtained.

### Evaluation of sensing performances

All experiments were performed at RT. A Xe lamp was used as the light source. The devices were prepared by connecting both ends of the pillar films to two Au wires with conductive silver paint. The as-prepared devices were put inside a sealed chamber with a quartz window. Electrical characterization was recorded with a Keithley 2602B source meter. The vapours of analytes were generated with their powder or liquid in a chamber. The saturated vapours were generated by blowing air through explosive powder.

### Computational method

BDC-NH_2_, a cluster model of (Ti_8_O_12_(HCOO)_12_) and nitro-explosives @BDC-NH_2_ were used to simulate the ligand of NH_2_-MIL-125, the node of NH_2_-MIL-125 and nitro-explosives adsorption on the ligand of NH_2_-MIL-125, respectively. DFT and time-dependent density functional theory (TD-DFT) calculations were performed on these models to obtain the ligand-localized excitations, node-localized excitations and the electronic structure properties of nitro-explosives binding with the ligand. A (TiO_2_)_6_ cluster model was used to simulate the TiO_2_ semiconductor. The ground-state geometries were fully optimized by using B3LYP functional with a def2-SVP basis set. All calculations were carried out using the Gaussian 09 package.

## DATA AVAILABILITY

All data reported in this study are available upon request by contact with the corresponding authors.

## Supplementary Material

nwac143_Supplemental_file
