# Supplementary material for: Non-contact real-time detection of trace nitro-explosives by MOF composites visible-light chemiresistor
Source: Natl Sci Rev. 2022 Jul 22;9(10):nwac143. doi: 10.1093/nsr/nwac143 (PMC9522384; doi:10.1093/nsr/nwac143)
Supplement: nwac143_Supplemental_file [file nwac143_supplemental_file.pdf]

Supporting Information

Non–Contact Real–Time Detection of Trace Nitro–Explosives by MOF Composites Visible–Light Chemiresistor

Wei–Hua Deng†, Ming–Shui Yao†, Min–Yi Zhang, Masahiko Tsujimoto, Kenichi Otake, Bo Wang, Chun–Sen Li*, Gang Xu*, and Susumu Kitagawa**

Correspondence to: bowang@bit.edu.cn, chunsen.li@fjirsm.ac.cn, gxu@fjirsm.ac.cn, kitagawa@icems.kyoto–u.ac.jp

This PDF file includes:

Materials
Methods
Experimental section
The limit of detection (LOD)
Computational Section
Figs. S1 to S29
Tables S1 to S6
References (41–53)

1. Materials

All chemicals and reagents were purchased from Alfa Aesar Co., China, Shanghai Chemical Reagent Co., China and Sigma–Aldrich. Nitrobenzene (NB) and 1.2–Dinitrobenzene (o–DNB) were purchased from Sigma–Aldrich. All chemicals were used without further purifications. Al_2O_3 substrates ($8 \times 20 \times 0.6$ mm^3) were purchased from Shenzhen Beilong Electronic Materials, China. Conductive silver paint (SPI# 05002–AB) was purchased from Structure Probe, Inc., USA. The analytic gases were purchased from Beijing Hua Yuan Gas Chemical Industry Co., Ltd., China. The disposable medical masks were purchased from Xiamenglanxing, Co., Ltd., China.

2. Methods

2.1 Growth of TiO_2 pillars on Al_2O_3 substrate.

Rutile TiO_2 pillars on Al_2O_3 substrate was prepared by a modified method reported (*41*). A TiO_2 seed layer was firstly formed on the substrate by thermally decomposing titanium n–butoxide ($Ti(OC_4H_9)_4$, TNB) at 450 ℃. Then, the seeded substrate was up–down immersed into the mixed solution of TNB (0.4 mL) and 6 M HCl (12 mL) in a 20 mL Teflon–lined autocalve. After reaction at 150 ℃ for 4 h, the reactor was cooled down to RT and TiO_2 pillars grown on Al_2O_3 substrate was obtained. The as–prepared TiO_2 pillars were rinsed by deionized (DI) water for 3 times. After drying, they were further annealed in air at 450 ℃ for 30 min.

2.2 Preparation of CSP (TiO_2, NH_2–MIL–125)

A two–steps seed–assisted solvothermal method was developed to grow a NH_2–MIL–125 sheath on the surface of TiO_2 pillars. Firstly, a piece of TiO_2 pillars was face–down immersed into a mixed solution containing 2–aminoterephthalate (BDC–NH_2) (0.1086 g, 0.6 mmol), dimethyl formamide (DMF, 9 mL) and methanol (1 mL) in a 20 mL Teflon–lined autoclave. The sealed autoclave was kept at 150 ℃ for 12 h, and then cooled down to RT. After being rinsed thoroughly with DMF and methanol, respectively, TiO_2 pillars was immersed into a mixed solution of TNB (0.104 mL, 0.3 mmol), DMF (9 mL) and methanol (1 mL) in a 20 mL Teflon–lined autoclave. The sealed autoclave was heated to 150 ℃ and kept for 4 h, and then cooled down to RT. After being rinsed with DMF and methanol, respectively, the MOF seeded TiO_2 pillars was obtained. The seeded sample was up–down immersed into mixed solution containing BDC–NH_2 (0.1086 g, 0.6 mmol), TNB (0.104 mL, 0.3 mmol), DMF (9 mL) and methanol (1 mL) in a 20 mL Teflon–lined autoclave and heated at 150 ℃ for 72 h.

After that, CSP (TiO_2, NH_2–MIL–125)–15nm was obtained and washed with DMF and methanol, respectively, and dried at 150 ℃ for 12 h.

2.3 Characterizations

Powder X–ray diffraction (PXRD) patterns were recorded on a Rigaku MiniFlex 600 diffractometer using Cu Kα radiation. The morphology and structure were observed by SEM (JEOL JSM–6700F) and TEM (Tecnai F20). UV–vis DRS spectra were collected on a Perkin–Elmer Lambda 950 spectrophotometer. $BaSO_4$ was used as a white standard.

2.4 Evaluation of sensing performances

All experiments were performed at RT. A Xe lamp (300 W) with a cut–off filter (420 nm < λ < 790 nm) was used as the visible light source and the monochromatic visible lights were obtained by using band–pass filters for the sensing measurements. The devices were prepared by connecting the both ends of the pillar films to two Au wires with conductive silver paint. The experiments were performed using a modified home–made chemiresistive sensor analysis setup reported previously (36) (**Fig. S2**). The as–prepared devices were put inside a sealed chamber with a quartz window. Electrical characterization was recorded with a Keithley 2602B source meter. The bias on the devices was set to be 5 V. The vapors of analytes were generated with their powder or liquid without heating in a chamber with the volume of ~ 26 mL. For the static gas sensing test, the powders of nitro–explosives (1.4 g RDX, 2 g TNT, or 2.4 g TNP) were kept at RT for 12 h to obtain saturated vapors. For the dynamic gas sensing test, dry air was used as the purging gas and carrier gas of analyte vapors / gases. The saturated explosive vapor (RDX, TNT or TNP) were generated by blowing air through 1.4 g RDX, 2.0 g TNT or 2.4 g TNP powder. Two mass flow controllers were used to accurately control the flows of the analyte vapors / gases and dry air, respectively, to obtain controlled concentrations of the target gases. The quartz chamber (10 ml) can be fulfilled in ~ 0.17 min at the total flow rate of 60 sccm.

The response (R) is defined as: $R = (R_{analyte} / R_{air} - 1) \times 100\%$ or $R = (R_{air} / R_{analyte} - 1) \times 100\%$, Where R_{air} and $R_{analyte}$ are the resistance of the devices in air and analyte, respectively. The response time of the sensor is the time required increasing the response to 90% of the saturation value and the recovery time is the time required decreasing the response to its 10%. The coefficient of variation (CV) is defined as: $CV = R_{SD} / R_{average} \times 100\%$, where R_{SD} and $R_{average}$ are the standard deviation (SD) and average value of responses with five successive cycles.

As shown in **fig. S15**, Molecular formulas and definition for nitro–explosives and some interference vapors used in this manuscripts (Trinitrophenol (TNP); trinitrotoluene (TNT);

Hexogeon (RDX); 1.2–Dinitrobenzene (o–DNB); nitrobenzene (NB); phenol (PhOH); hydroquinol (HQ); o–nitrophenol (ONP))

2.5 Computational method

BDC–NH_2, a cluster model of [$Ti_8O_{12}(HCOO)_{12}$] and nitro–explosives @BDC–NH_2 [including TNP@BDC–NH_2, RDX@BDC–NH_2, TNT@BDC–NH_2, and o–DNB@BDC–NH_2] were used to simulate the ligand of NH_2–MIL–125, node of NH_2–MIL–125, and nitro–explosives adsorption on ligand of NH_2–MIL–125, respectively (Some non–nitro sensors of PhOH, acetone, toluene, and benzene also have been studied for comparison, all the optimized structures see the **Fig. S23**). Density functional theory (DFT) and time–dependent density functional theory (TD–DFT) calculations were performed on these models to obtain the ligand–localized excitations, node–localized excitations, and the electronic structure properties of nitro–explosives binding with ligand. A $(TiO_2)_6$ cluster model was used to simulate the TiO_2 semiconductor, as the $(TiO_2)_6$ cluster has been reported that it is a smallest and reliable TiO_2 model for TiO_2 semiconductor (*42, 43*). The ground–state geometries were fully optimized by using B3LYP functional with def2–SVP basis set. All calculations were carried out with Gaussian 09 package (*44*). Furthermore, the charge transfer integral (J_{eff}) between ligand and nitro–explosive in an inner couple within the dimer of nitro–explosives binding with ligand was estimated following the so–called projective method, using the NWchem program (*45*).

3. Experimental Section

3.1 Caution: TNT, TNP, RDX and other explosives used in the present study are highly explosive and should be handled only in small quantities (46).

3.2 Explosive detection:

The experiments were performed using a home–made setup (**Fig. S2**). The as–prepared CSP (TiO_2, NH_2–MIL–125) devices were put inside a sealed chamber and dry air was used as carrier gas. The RDX vapor was generated from1.4 g RDX powder in a quartz tube with a volume of 10 mL. All experiments were performed at RT under visible light.

3.2.1 Static–state experiments

As shown in **Fig. S2**, the explosive powder (2.0 g TNT, 2.4 g TNP or 1.4 g RDX) was placed in chamber B and firstly heated overnight at 80 ℃ in flowing air to remove the small amount of moisture in it. Then, the sensing devices were put in the quartz chamber C. After that, the valve a, b and d were closed, and the chamber B was connected with chamber C through three–way valve c. The setup was kept at RT under dark for 48 h to form a saturated

vapor atmosphere in the chamber C. Next, the current (I_e) under the saturated explosive vapor was recorded under visible light irradiation. The visible light was generated by a Xe lamp and a cut–off filter (420 nm < λ < 790 nm). After that, the valve b was turned off and the valve a and d were turned on. The chamber C was connected with the chamber A through valve c. Dry air was used to purge the chamber C. Whereafter, the valve a, b and d were closed, the current under the static air (I_a) was recorded under visible light. The sensor response in static–state experiments is defined as, Response = $(I_a – I_e)/I_e \times 100\%$, where I_e and I_a are the current of the sensor in explosive vapor and in dry air, respectively.

The procedures and conditions of static distance–dependent tests were similar to above mentioned experiments, while by changing the distance between RDX and the sensor. The exposure time to static state RDX vapor is 1 h.

3.2.2 Dynamic–state experiments

As shown in **Fig. S2**, explosive powder (2.0 g TNT, 2.4 g TNP or 1.4 g RDX) was placed in chamber B and firstly heated overnight at 80°C in flowing air to remove the small amount of moisture in it. Mass flow controllers (MFC) with control units used to accurately control the explosive vapor flows. At the beginning of the experiment, Xe lamp was turned on. The valve b was turned off and the chamber C was connected with the chamber A through valve c. The test chamber was purged with dry air for 30 min to ensure the absence of water vapor and also to establish the baseline, the current under the dry air (I_a) was recorded under visible light and at RT. At the same time, explosive (RDX, TNT or TNP) saturated vapor atmosphere was formed in the chamber B. The preconcentration of RDX vapor took place in an oil bath at 150°C for 12 h. The equilibrium vapor concentration gradient of the RDX was realized by gradient reduction of oil bath temperature. The oil bath temperature is set as 150, 130, 110, 90, 70, 50, 25°C with respective equilibrium time of 50 min. The low temperature equilibrium vapor concentration gradient of the RDX was realized by moving the chamber B to a chiller (DLSB–5/20, Zheng zhou Great Wall Scientific industrial and Trade Co., Ltd., China) with gradient reduction of solvent bath temperature (253 K –293 K, Table S1). The devices were measured at room temperature under visible light. The measurements were conducted in 10 mL quartz container with dry air flow of 60 sccm.

The current under different vapor atmosphere (I_e) was recorded under visible light and at RT. It should be noted that the CSP (TiO_2, NH_2-MIL-125) pillars are connected with each other to form a continuous conductive network, instead of the perfect vertical nanowires that separated from each other. As shown in **Fig. S2**, silver paste was painted on the top of CSP (TiO_2, NH_2-MIL-125) and also sank into its bottom, and then connected with a pair of Au

wires for the control and readout of DC voltage/current signals. The charge carrier transported along the nanowires and their inter-connected parts, of which the resulted current was recorded *via* electrodes using Keithley 2602B Source meter.

For distance dependent dynamic test, after heating RDX vapor at 150°C, the chamber B was cooled to preconcentrate the saturated vapor pressure of RDX at RT. Other procedures and conditions are the same as above dynamic test.The mass–dependent dynamic tests were conducted with similar procedures and conditions as above dynamic test, while by changing the mass from 5 mg to 1.4 g. The chamber B was kept at 150°C for preconcentration of RDX vapor during the whole test.

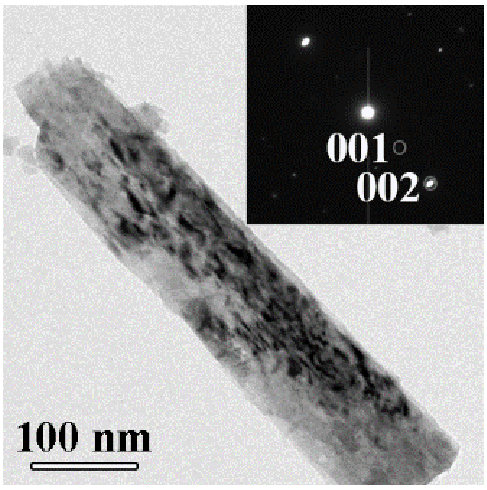

Fig. S1. TEM image and corresponding selected–area electron diffraction (SAED) pattern of TiO_2 nanowire.

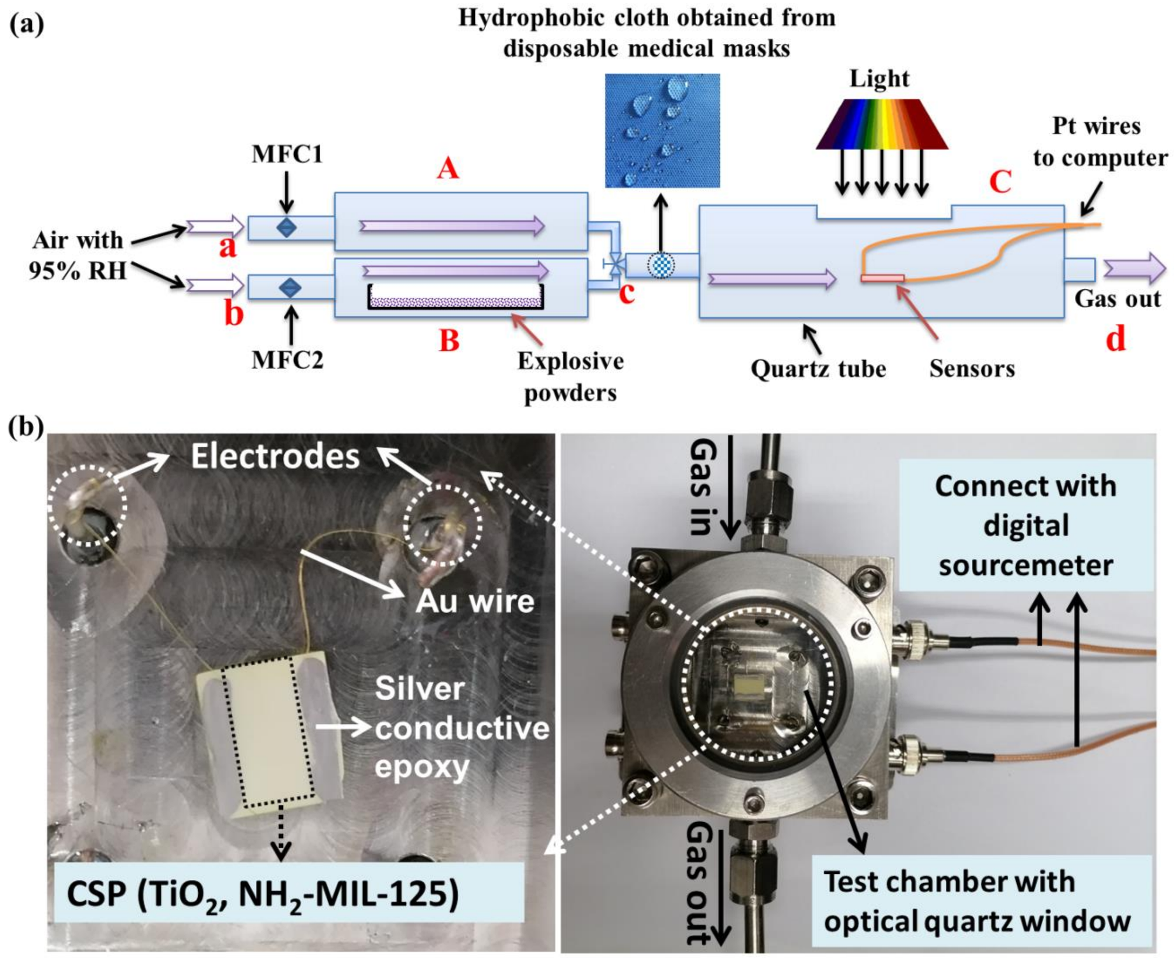

Fig. S2. (a) Schematic illustration of the explosive–detection setup under visible light; (b) Digital photographs of the sensing devices and the sensing setup. The device was manually wired to the electrical wires by silver paste. CSP (TiO_2, NH_2–MIL–125) on Al_2O_3 substrate was prepared by our developed two–step seed–assisted solvothermal method. 20 pieces would be fabricated in every batches as needed, and, for the record, it is easily expanded for real–world applications.

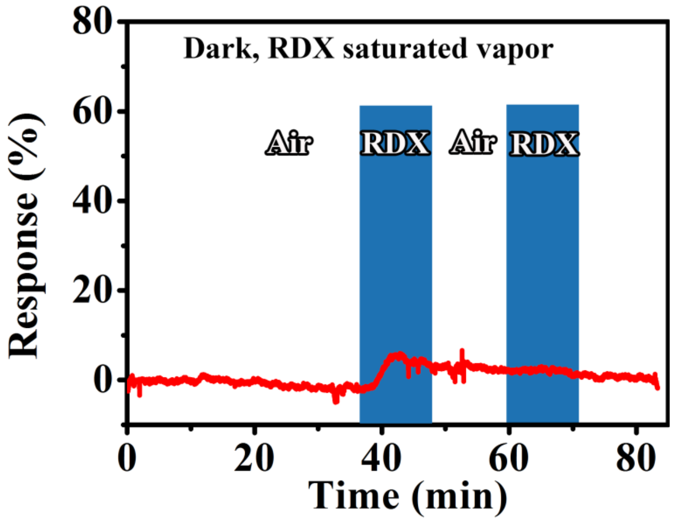

Fig. S3. Dynamic response–recovery curve of CSP (TiO_2, NH_2–MIL–125) toward saturated RDX vapors under dark at RT.

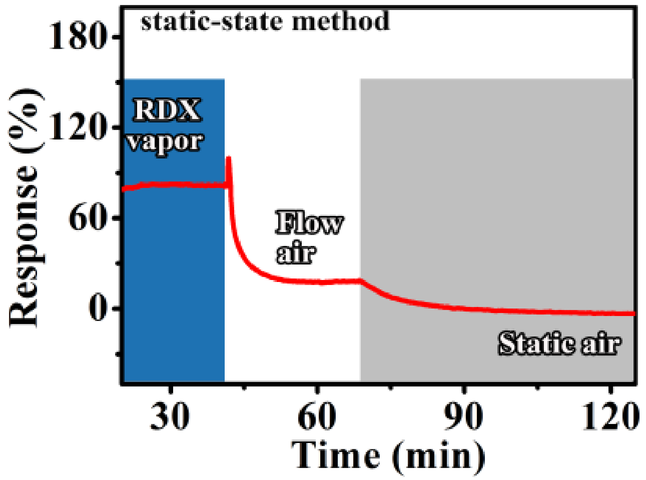

Fig. S4. Static–state response–recovery curve of CSP (TiO_2, NH_2–MIL–125) toward saturated vapor of RDX under visible light. The current of CSP (TiO_2, NH_2–MIL–125) device in static RDX saturated vapor was monitored. After that, the chamber was cleaned by flowing air and a static pure air atmosphere was created. The static–state response of CSP (TiO_2, NH_2–MIL–125) was deduced as the current difference in static saturated RDX vapor and air.

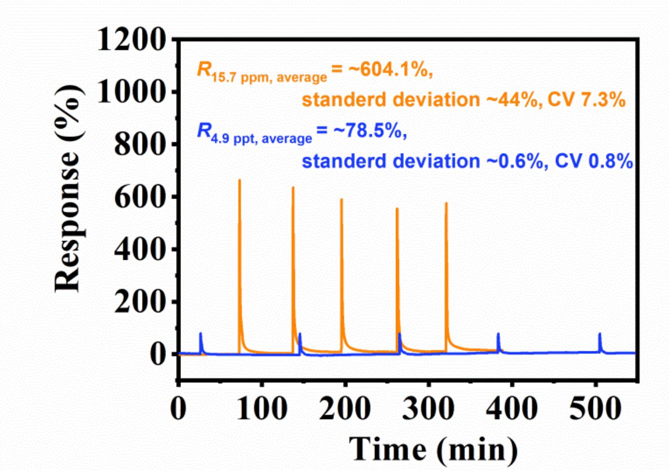

Fig. S5. Dynamic response–recovery successive cycles curves for 4.9 ppt (blue line) and 15.7 ppm of RDX (orange line).The average response value of 78.8% is close to the value obtained in the static test (82%).

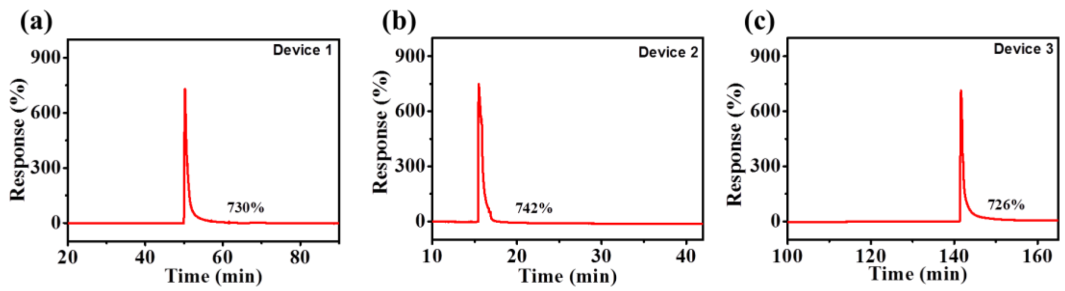

Fig. S6. Real–time response–recovery curves of three different CSP (TiO_2, NH_2–MIL–125) sensors fabricated in different batches upon exposure toward 15.7 ppm RDX under visible light, the response are 730%, 742% and 726%, respectively. The CV (coefficient of variation) is only 1.1%, which showed good reproducibility.

4. The limit of detection (LOD)

Since there is no standard method for the theoretical LOD estimation for chemiresistive gas sensor due to its power–law govern response vs. concentration, the 3RMS/gradient rule based LOD calculation is not applicable for RDX in this work. In contrast, the experimentally measured LOD is more reliable. We have directly measured the response to lower concentration RDX vapor which is obtained by controlling the evaporation temperature of RDX at 253 K (**Fig. S7**). Notably, a noticeable response to 8×10^{-4} ppt was obtained. The R = 10% based LOD calculation for TNT and TNP is just used to facilitate a better understanding of researchers from other research areas.

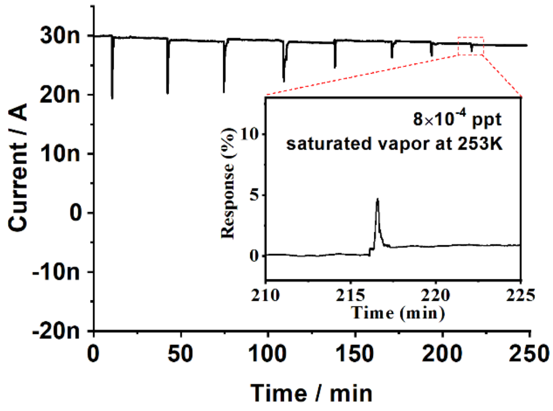

Fig. S7 Dynamic response–recovery curve of CSP (TiO_2, NH_2–MIL–125) sensor toward RDX vapor preconcentrated at low temperature (293 K–253 K, step: 5 K, the inset is the corresponding response curve toward RDX vapor preconcentrated at 253 K, the expected saturated concentration is 0.0008 ppt or 0.0146 ppt, according to *J. Energ. Mater.,1986, 4, 447 (30)* and *J. Phys. Chem. A*. 2021, 125, 1279 (*29*), respectively).

Table S1. Calculated saturated concentration of RDX vapor preconcentrated at different temperature.

T/K	J. Energ. Mater., 1986, 4, 447	J. Phys. Chem. A. 2021, 125, 1279
253	0.000822	0.014598
258	0.002575	0.035171
263	0.007724	0.08287
268	0.022234	0.191109
273	0.061571	0.431704
278	0.164372	0.955967
283	0.423846	2.076654
288	1.057561	4.428413
293	2.557701	9.276391

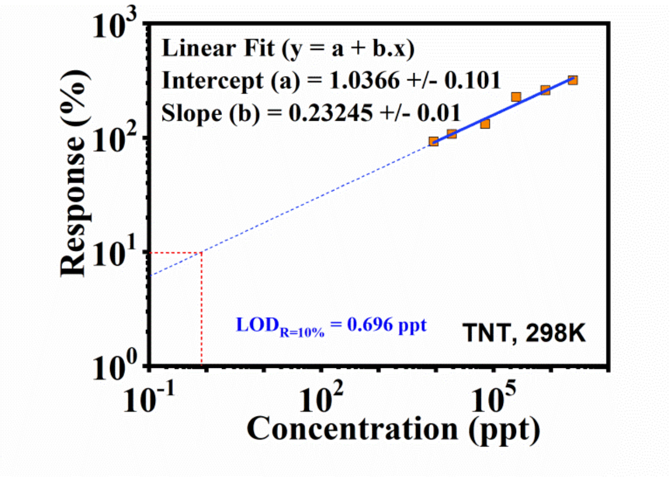

Fig. S8. Linear fitted plots of response *vs* TNT concentration.

Table S2. Calculation of sensitivity, RMS_{noise} and LOD of CSP (TiO_2, NH_2–MIL–125) sensor toward TNT vapor.

a	Slope b (ppt^{-1})	Standard Error (ppt^{-1})	LOD (ppt)	Method
1.0366	0.23245	0.01	0.696	R=10%

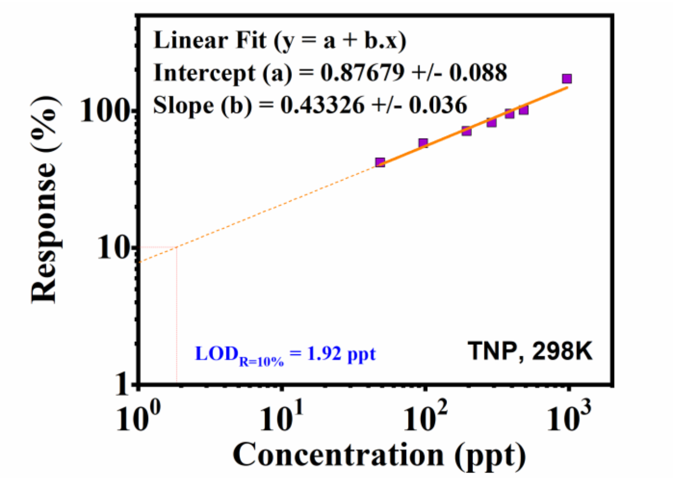

Fig. S9. Linear fitted log–log plots of response *vs* TNP concentration and the corresponding LOD calculations.

Table S3. Calculation of sensitivity, RMS_{noise} and LOD of CSP (TiO_2, NH_2–MIL–125) sensor toward TNP vapor.

a	Slope b (ppt^{-1})	Standard Error (ppt^{-1})	LOD (ppt)	Method
0.87679	0.43326	0.036	1.92	R=10%

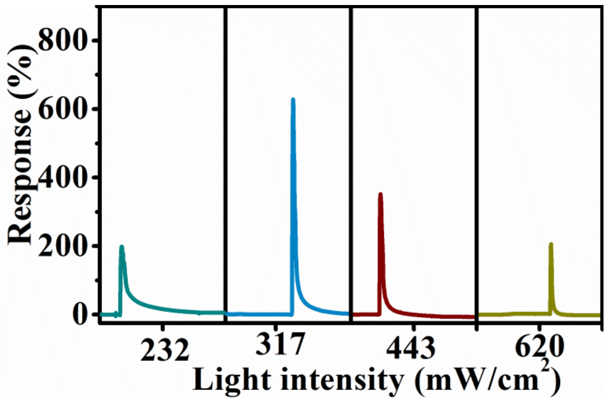

Fig. S10. Light intensity dependent responses of CSP (TiO_2, NH_2–MIL–125) to saturated RDX vapor. The highest response of the device was found at about 300 mW cm^{-2}.

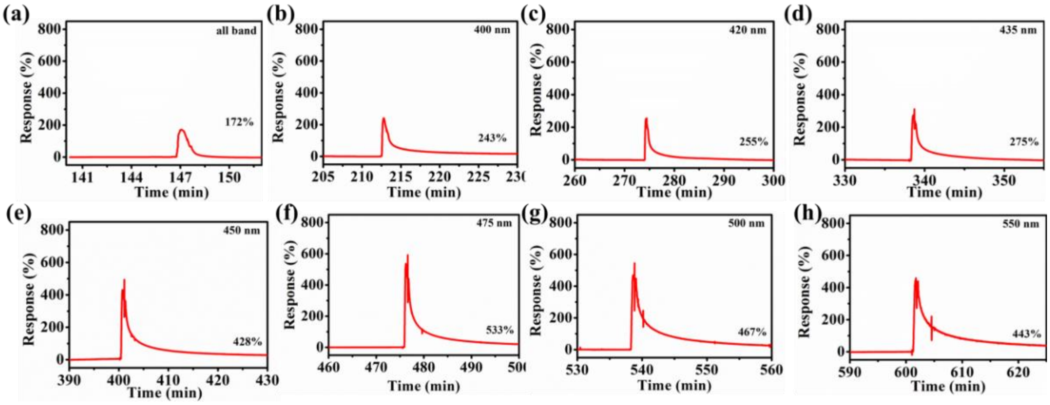

Fig. S11. Wavelength dependent responses of CSP (TiO_2, NH_2–MIL–125) to 15.7 ppm RDX vapor. The highest response of the device was found at 475 nm.

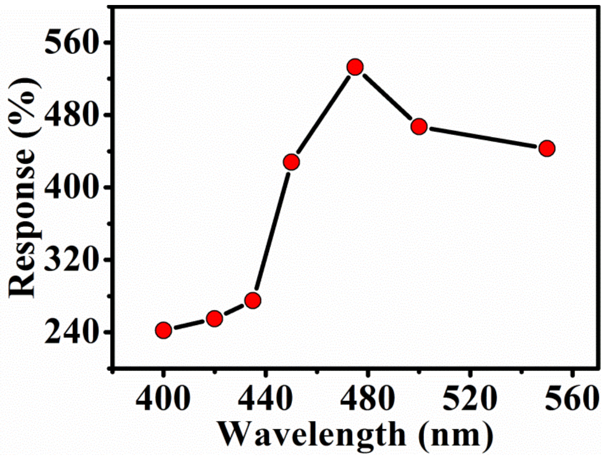

Fig. S12. Wavelength dependent responses of CSP (TiO_2, NH_2–MIL–125) deduced from Fig. S11. The energy of the absorbed light is decided by the light power density and wavelength. The higher energy of the absorbed light creates the higher density of photo–generated charge carriers and thus results the higher response. However, the higher energy density of the absorbed light also increases the desorption speed of RDX from sensing material and induces lower response (*37*). So the observed highest response of CSP (TiO_2, NH_2–MIL–125) to RDX is a balance in above competitive processes.

Table S4: The light intensity of the light source in Fig. S11 and S12

Wavelength (nm)	Light intensity (mW cm^{-2})
All band	378 $\pm$ 5
400	59 $\pm$ 5
420	50 $\pm$ 5
435	55 $\pm$ 5
450	53 $\pm$ 5
475	60 $\pm$ 5
500	55 $\pm$ 5
550	57 $\pm$ 5

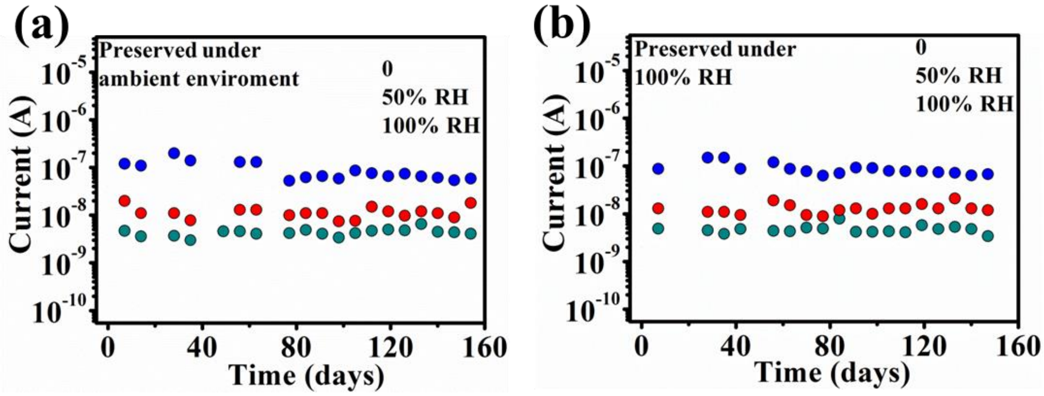

Fig. S13. Long–term baseline stability of CSP (TiO_2, NH_2–MIL–125) under ambient (a) and 100% RH conditions (b), respectively.

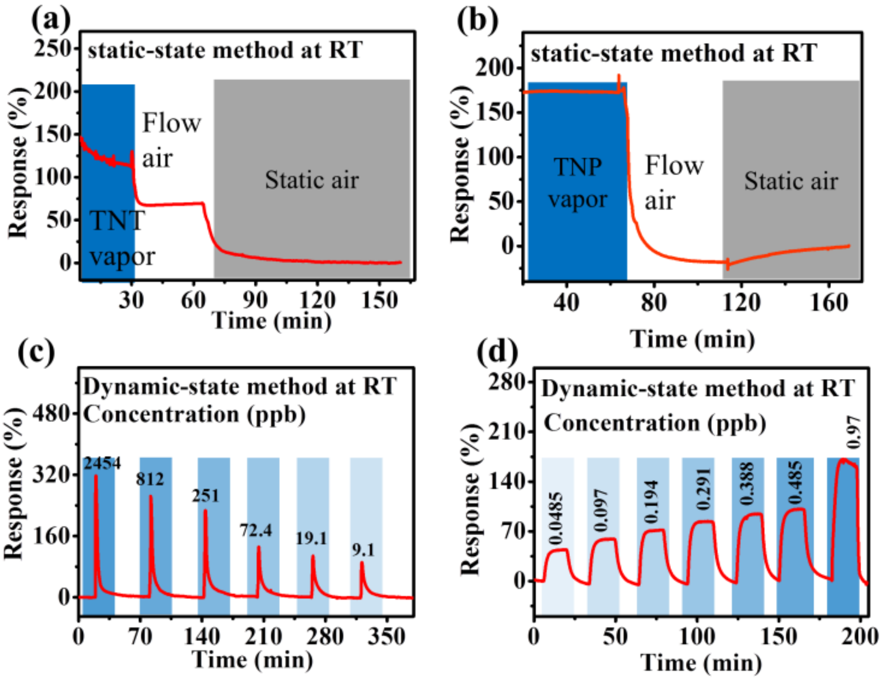

Fig. S14. Static–state response–recovery curve of CSP (TiO_2, NH_2–MIL–125) toward saturated vapor of (a) TNT and (b) TNP under visible light. Dynamic response–recovery curves for (c)TNT and (d)TNP at different expected concentrations under visible light.

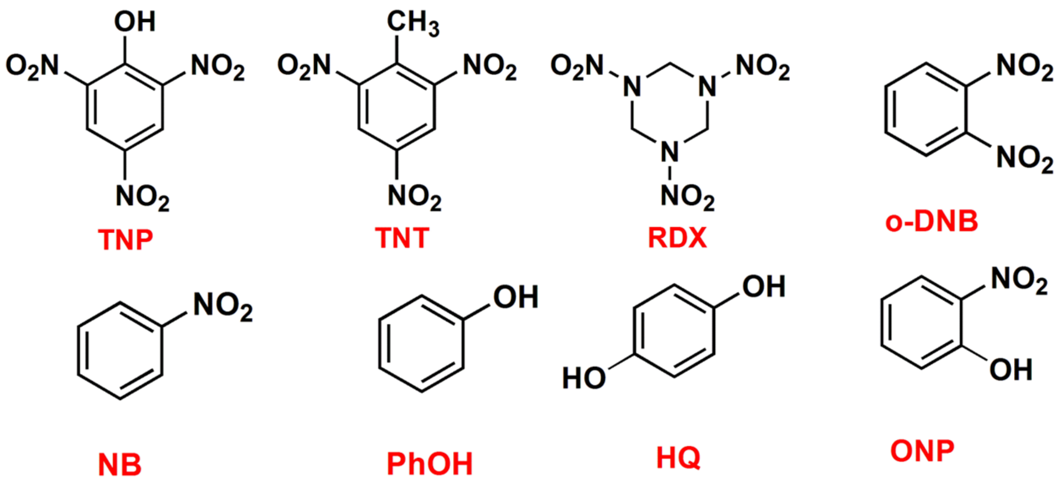

Fig. S15 Molecular formulas and definition for nitro–explosives and some interference vapors used in this manuscripts (Trinitrophenol (TNP); trinitrotoluene (TNT); Hexogeon (RDX); 1.2–Dinitrobenzene (o–DNB); nitrobenzene (NB); phenol (PhOH); hydroquinol (HQ); o–nitrophenol (ONP)).

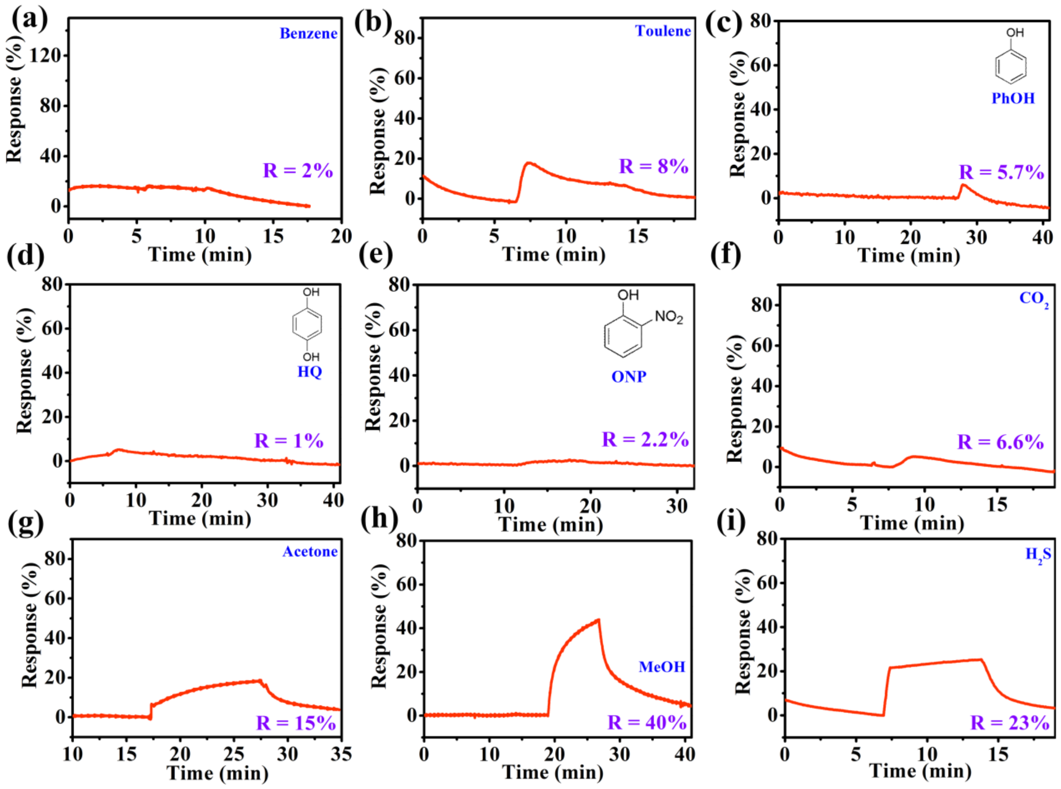

Fig. S16. Dynamic response curves of CSP (TiO_2, NH_2–MIL–125) sensor towards (a) 100 ppm benzene, (b) 100 ppm toulene, (c) saturated vapor of PhOH, (d) saturated vapor of HQ, (e) saturated vapor of ONP, (f) 100 ppm CO_2, (g) 100 ppm acetone, (h) 100 ppm MeOH, and (i) 100 ppm H_2S under visible light at RT.

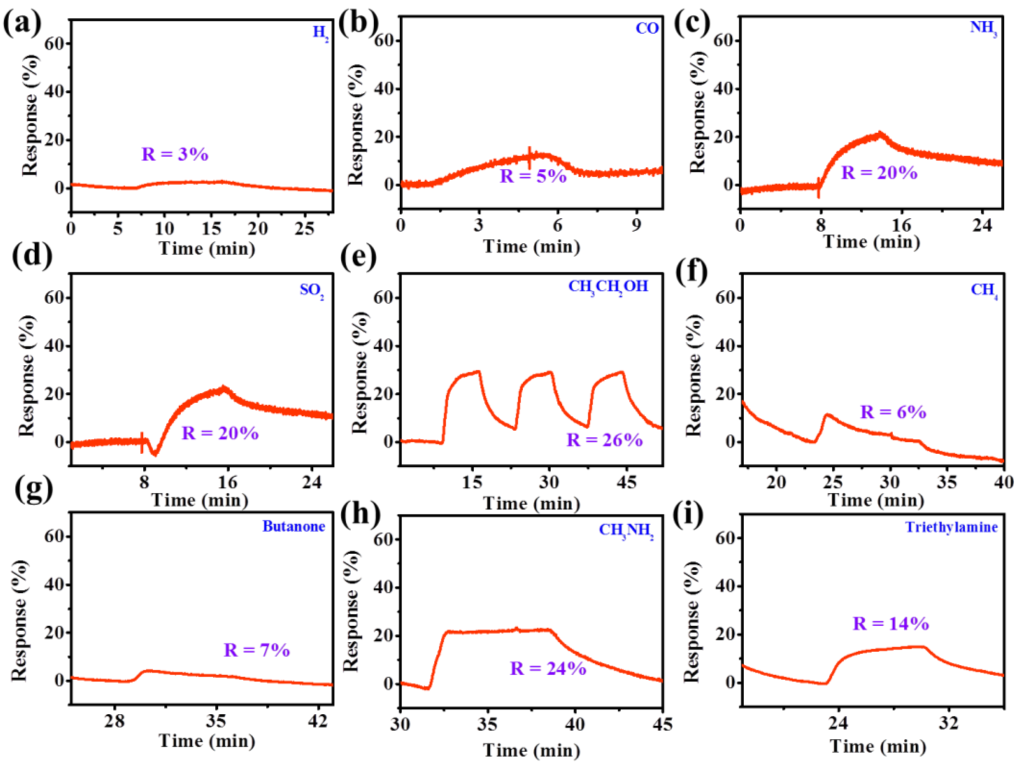

Fig. S17. Dynamic response curves of CSP (TiO_2, NH_2–MIL–125) sensor towards (a) 100 ppm H_2, (b) 100 ppm CO, (c) 100 ppm NH_3, (d) 100 ppm SO_2, (e)100 ppm CH_3CH_2OH, (f) 100 ppm CH_4, (g) 100 ppm butanone, (h) saturated vapor of CH_3NH_2, and (i) saturated vapor of triethylamine under visible light at RT.

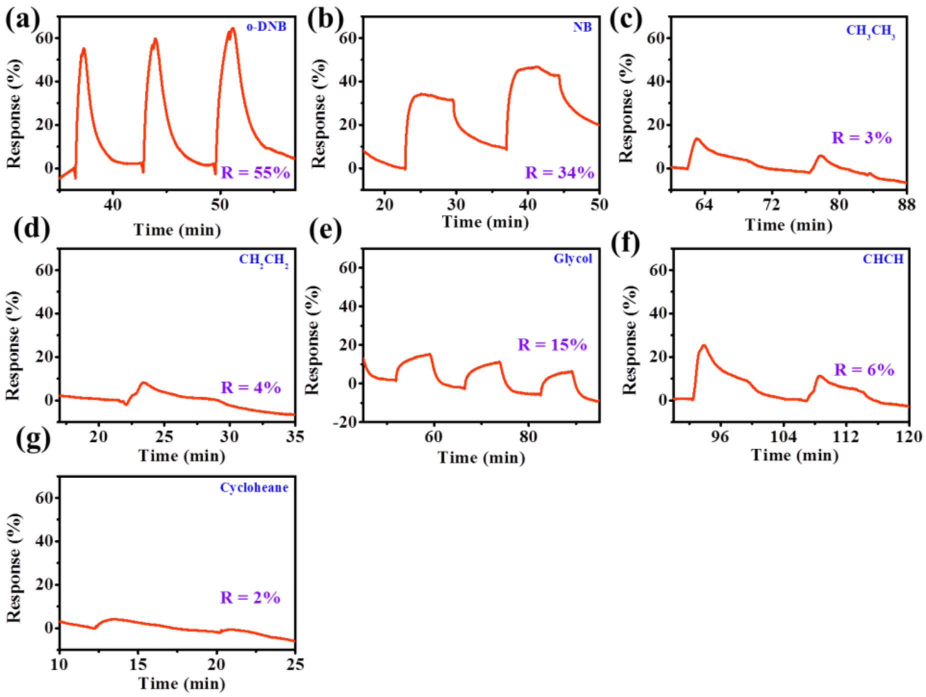

Fig. S18. Dynamic response curves of CSP (TiO_2, NH_2–MIL–125) sensor towards:(a) saturated vapor of o–DNB (b) saturated vapor of NB, (c) 100 ppm CH_3CH_3,(d) 100 ppm CH_2CH_2, (e)saturated vapor of glycol, (f) 100 ppm CHCH, and (g) saturated vapor of cyclohexane under visible light at RT.

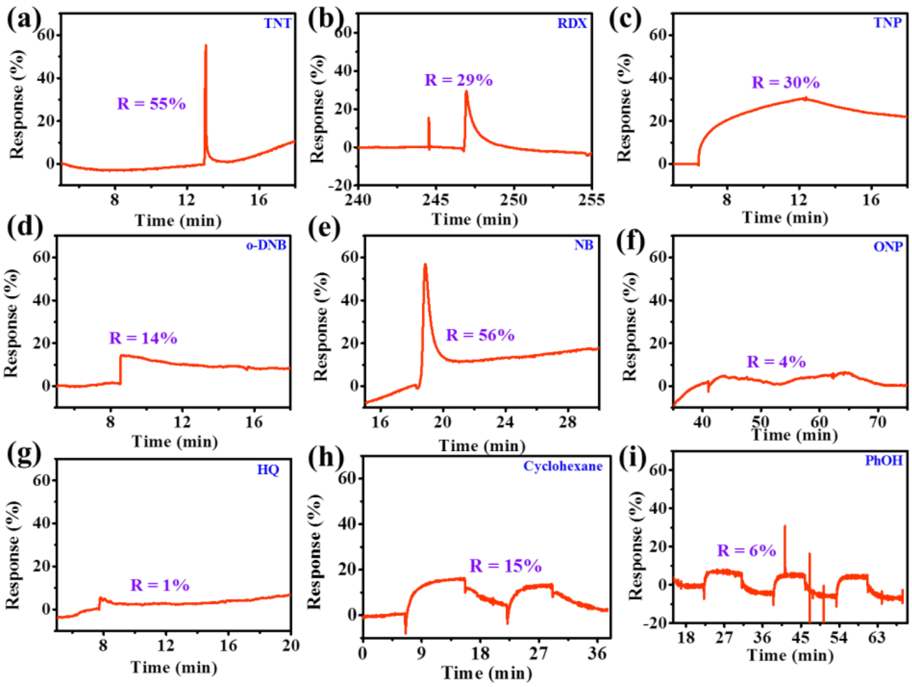

Fig. S19. Dynamic response curves of TiO_2 sensor towards saturated vapor of (a) TNT (b) RDX, (c) TNP, (d) o–DNB, (e) NB, (f) ONP, (g) HQ, (h) cyclohexane, and (i) PhOH under visible light at RT.

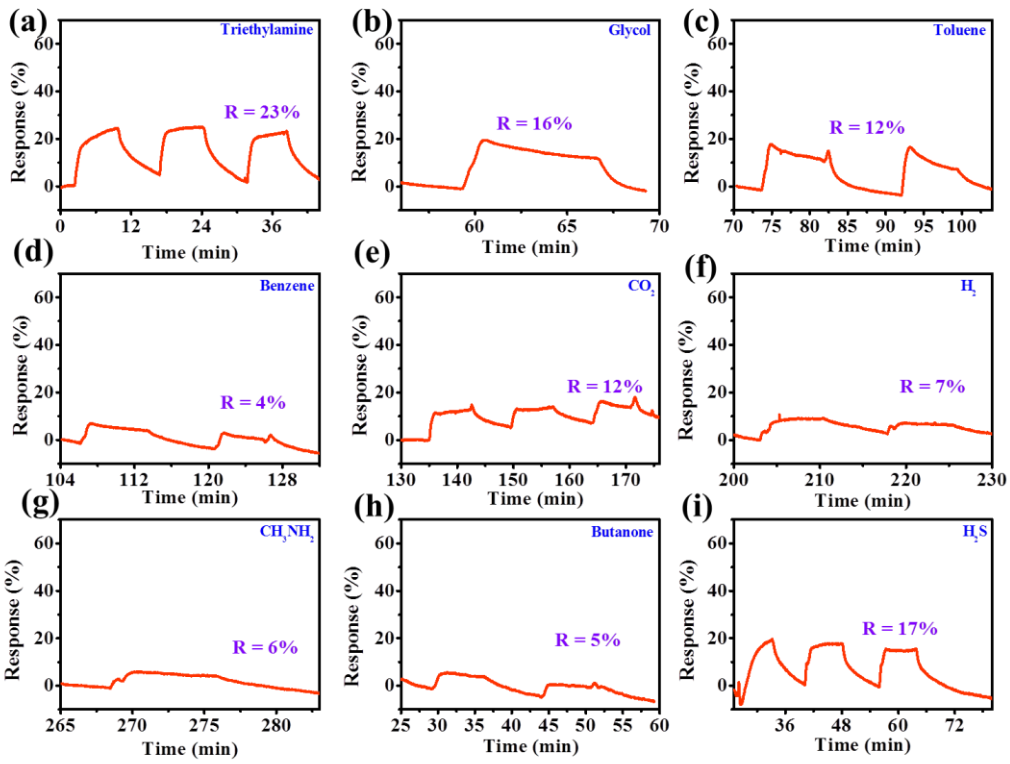

Fig. S20. Dynamic response curves of TiO_2sensor towards (a) saturated vapor of triethylamine, (b) saturated vapor of glycol, (c) saturated vapor of toluene, (d) 100 ppm benzene, (e) 100 ppm CO_2 (f) 100 ppm H_2, (g) saturated vapor of CH_3NH_2, (h) 100 ppm butanone, and (i) 100 ppm H_2S under visible light at RT.

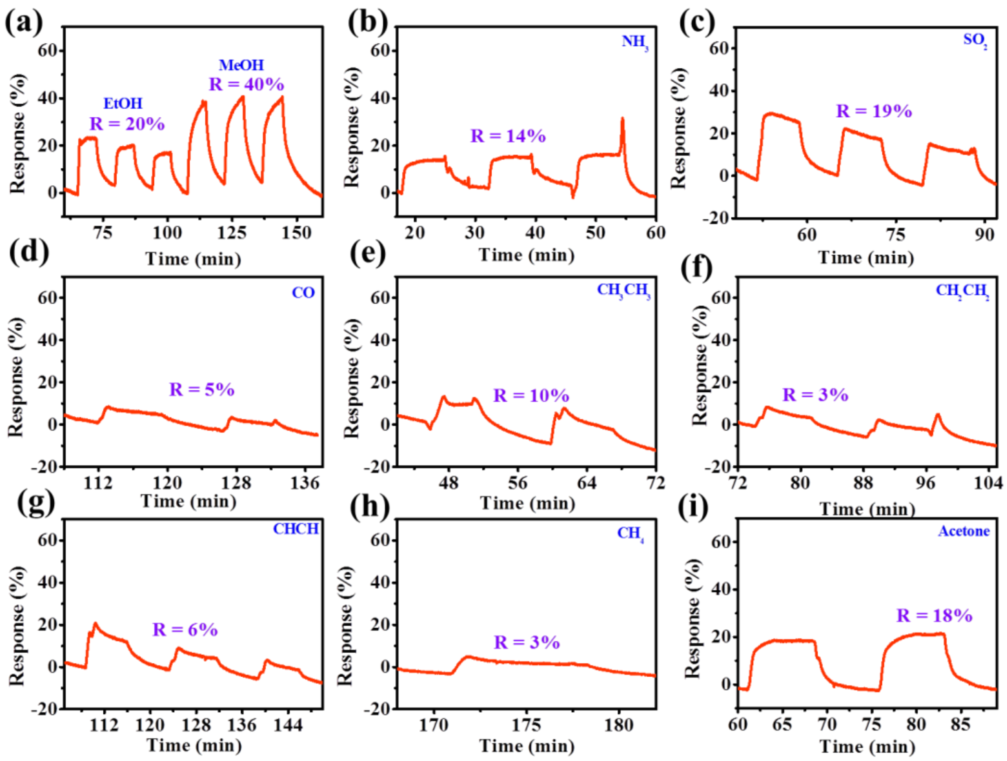

Fig. S21. Dynamic response curves of TiO_2sensor towards (a) 100 ppm EtOH and MeOH, (b) 100 ppm NH_3, (c) 100 ppm SO_2, (d) 100 ppm CO, (e) 100 ppm CH_3CH_3 (f) 100 ppm CH_2CH_2, (g) saturated vapor of CHCH, (h) 100 ppm CH_4, and (i) 100 ppm acetone under visible light at RT.

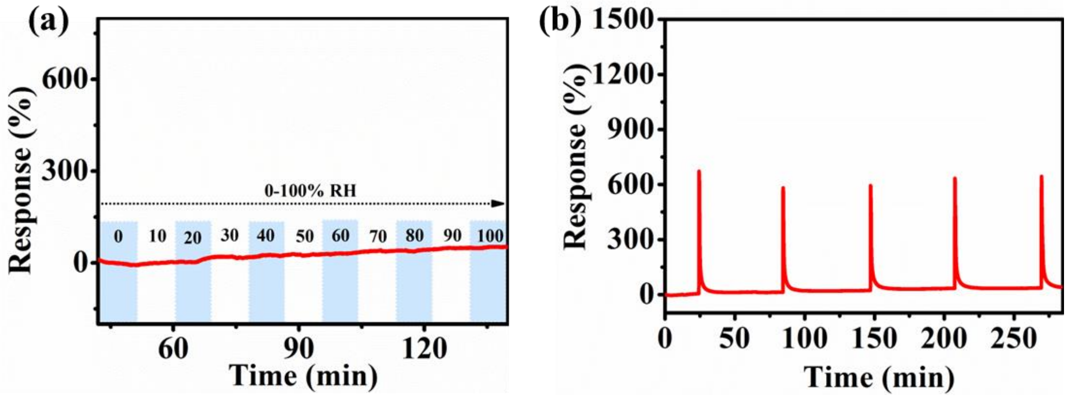

Fig. S22. (a) Real–time dynamic response–recovery curve in the humidity range of 0 – 100% RH under visible light (b) Real–time response–recovery curve of successive cycles toward 15.7 ppm RDX when using air with 95% RH as the carrier gas and by simply filtering the analyte vapor with 3 layers of the hydrophobic cloth obtained from disposable medical mask;.

5. Computational Section

The nitro–explosives adsorption on MOF (NH_2–MIL–125) system was investigated. As shown in **Fig. S23**, for simplification, the MOF (NH_2–MIL–125) system was simulated by a cluster models of [$Ti_8O_8(OH)_4(HCOO)_{12}$–Ligand–$Ti_8O_8(OH)_4$ $(HCOO)_{12}$], which two side of ligand(BDC–NH_2) were binding with the metal–oxo cluster of [$Ti_8O_8(OH)_4$ $(HCOO)_{12}$]with terminated by H atom.Moreover, the different studied nitro–explosive binding with the ligand also have been investigated, and their optimized structures show in **Fig. S24**. All ground–state geometries of the studied conformers were fully optimized at hybrid DFT level by B3LYP functional combined with Def2–SVP basis set.

The adsorption energies (**E**$_{ads}$) were obtained using the equation (*47*):

$$\mathbf{E}_{ads}=\mathbf{E}_{total}-\mathbf{E}_{adsorbate}-\mathbf{E}_{adsorbent} \quad \text{(S3)}$$

Where **E**$_{total}$ was the total energy of adsorption system, **E**$_{adsorbate}$ was energy of adsorbate, **E**$_{adsorbent}$ was the energy of $Ti_2O_2(OH)_6$–Ligand–@$(TiO_2)_6$ complex. According to the expression, the negative **E**$_{ads}$ indicates the absorption system is stable.

The relationship between charge–transport properties and geometric structure of nitro–explosives@BDC–NH_2 dimers can be understood by considering the electronic Hamiltonian in a simple tight–binding approximation (47):

$$\hat{H} = \sum E_i(\theta) a_i^+ a_i + \sum_{i \neq j} J_{i,j}(\theta) a_i^+ a_i \quad (1)$$

where, a_i^+ and a_i are creation and annihilation operators, $E_i(\theta)$ is site energy (energy of electron or hole at i^{th} molecular site) which is computed from the diagonal matrix element of Kohn–Sham (KS) Hamiltonian, $E_i = \left\langle \varphi_i \middle| \hat{H}_{KS} \middle| \varphi_i \right\rangle$. Second term of equation(1) is charge transfer integral $(J_{i,j})$ which is computed from the off–diagonal matrix element of the KS Hamiltonian, $J_{i,j} = \left\langle \varphi_i \middle| \hat{H}_{KS} \middle| \varphi_j \right\rangle$. In principle, $J_{i,j}$ provides the strength of electronic coupling between φ_i and φ_j (HOMO or LUMO of nearby molecules i and j)which relates the superposition nature of neighboring electronic states. The charge transfer rate (*k*) is calculated by using semi–classical Marcus theory, and is defined as (*48–50*).

$$k = \frac{J_{eff}^2}{\hbar} \sqrt{\frac{\pi}{\lambda k_B T}} \exp\left[-\frac{\left(\Delta E_{ij} - \lambda\right)^2}{4\lambda k_B T} \right] \quad (2)$$

where, J_{eff}, $\hbar$, k_B, λ and T are effective charge transfer integral, reduced Planck constant, Boltzmann constant, reorganization energy and temperature, respectively. ΔE_{ij} is site energy difference between nearby electronic states. Commonly site energy differences are influenced by applied electric field, electrostatic interaction and polarization. In this work, we only consider the J_{eff} to understand the charge transfer in between the ligand and nitro–, since it is enough to judging the possible formation of charge transfer compound from the ligand and sensor, and it is more complex to the evaluate other parameter in the MOF system with consist of metal–oxo node and ligand.

The J_{eff} is defined in terms of charge transfer integral (J), spatial overlap integral (S), site energies of adjacent i^{th} and j^{th} sites (E_i and E_j) and is expressed as (*51–53*)

$J_{eff} = J_{i,j} - S_{i,j}\left(\frac{E_i + E_j}{2}\right)$, where, $S_{i,j}$ is the spatial overlap integral of nearby sites $S_{i,j} = \left\langle \varphi_i \middle| \varphi_j \right\rangle$.

The above charge transport key parameters J, E and S are calculated using fragment molecular orbital approach as employed in nwchem program.

In MOF sheath, our density functional theory(DFT) calculations (**Fig.4c, 4d, S23** and **Table S5**) revealed that, the LUMO level of ligand (BDC–NH_2) is –1.95 eV, which is higher than the LUMO of inorganic $Ti_8O_8(OH)_4(HCOO)_{12}$ node (–3.30 eV). As results, the photo–excited electrons on the ligand are thermodynamically favored to transfer to the inorganic node through a ligand–to–metal charge transfer (LMCT) process. Subsequently, these electrons would randomly transfer to the HOMO of 12 ligands on the inorganic node and 1/3 of them diffuse towards to MOF/TiO_2 interface. Notably, the conduction band of TiO_2 (–4.22 eV) is more negative than the LUMO of inorganic node (–3.30 eV) in MOF, the photogenerated electrons would be induced and trend to transfer to MOF–TiO_2 interface. Therefore, the above mentioned ratio is an underestimated value. At the MOF–TiO_2 interface, the LUMO of inorganic node (–3.30 eV) and the HOMO of ligand (–5.91 eV) in MOF are above the conduction band (–4.22 eV) and valence band (–7.86 eV) of TiO_2, respectively. Consequently, a staggered–gap (type II) heterojunction was created at MOF–TiO_2 interface (**Fig. 1**), known to be thermodynamically favored in promoting the separation and reducing the recombination of the photogenerated charge carriers, is created at MOF–TiO_2 interface. Thus, it can act as a “pump” to extract the photo–excited electrons generated at both MOF sheath and MOF–TiO_2 interface to TiO_2 to produce active oxygen species for sensing reaction (*27*). More interestingly, an unexpected analyte self–promoted sensing behavior was revealed. It is calculated that the nitro–explosives molecules are favor to binding with the BDC–NH_2 ligand, since the binding energies of TNP@ligand, TNT@ligand, RDX@ligand and o–

DNB@ligand are –7.91, –8.11, –8.09 and –8.73 kcal/mol (see **Table S5**). Moreover, the adsorption of these nitro–explosives molecules could induce great changes in electronic structures of MOF. Firstly, these dimers of nitro–explosives molecules binding to ligand of MOF have much lower HOMO–LUMO gaps than that of ligand (see **Table S5**), which could extend the light absorption range. Secondly, as shown in **Fig. S23**, the HOMO orbitals of nitro–explosives@BDC–NH_2 compounds all highly locate on the BDC–NH_2. In contrast, their LUMO orbitals highly locate on their nitro–explosive molecules separately, which indicate that there is strong charge transfer from the ligand to nitro–explosive molecules within these compound under the visible lighting. In contrast, as the interferences (PhOH, acetone, toluene, and benzene in our calculations) bind to BDC–NH_2 ligand to form the compounds, both of their HOMO and LUMO orbitals are locate on the BDC–NH_2 ligand, which imply no charge transfer from ligand to them. Therefore, nitro–explosives adsorb on ligand of MOF could also promotes the separation and transfer of the photo–excited charge carriers from MOF to the interface (**Fig.4d**).

Take the RDX@ligand model as an example, its HOMO totally locate on the ligand part with energy level of –5.49 eV slight higher than that of ligand, while its LUMO orbital totally locates on the RDX part and down–shift the LUMO level from –1.95 eV of ligand to –2.65 eV of RDX@ligand. This new band structure not only results in a smaller energy gap than that of ligand and would extend the light absorption range, but more importantly, would further promotes the photo–excited electrons transfer from MOF to the interface. The calculated charge transfer integral (J_{eff}) of RDX@ligand on the HOMO to LUMO charge transfer pathway is 10 meV (See **Table S6**). This large J_{eff} value indicates that RDX adsorption would bring a faster electron transfer from ligand part to RDX part under the visible lighting, thus produce more photo–excited charges. Above extended light absorption range and accelerated generation, separation and transport of photo–excited electron owing to the binding of RDX are expected to promote the electron injection to enhance the sensing activity.

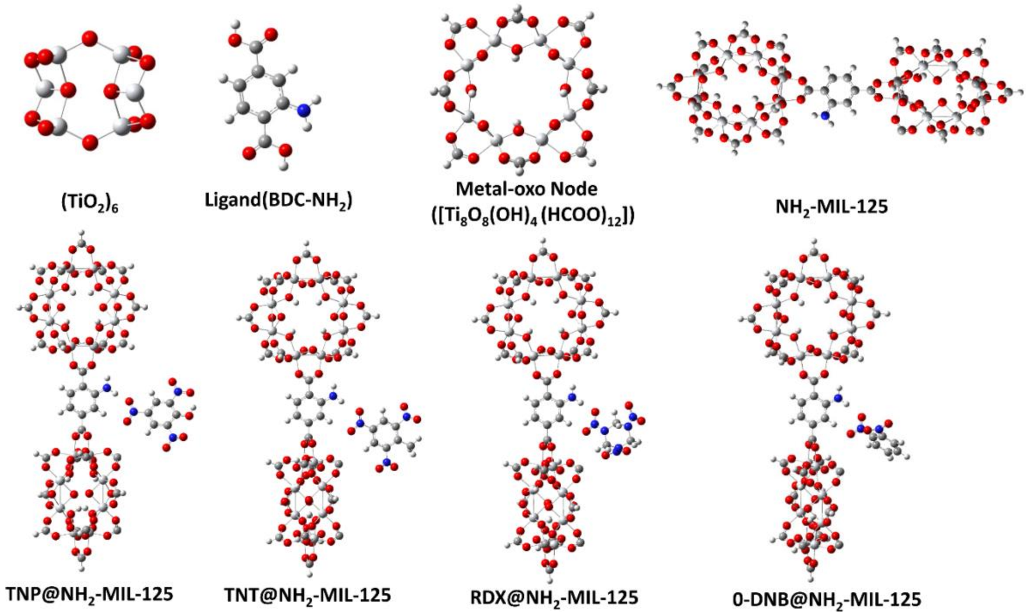

Fig. S23. Optimized geometric structures of $(TiO_2)_6$ cluster, Ligand(BDC–NH_2), metal–oxo node[$Ti_8O_{12}(HCOO)_{12}$] cluster, NH_2–MIL–125([$Ti_8O_{12}(HCOO)_{12}$]–Ligand–[$Ti_8O_{12}(HCOO)_{12}$] cluster), and nitro–explosives@ NH_2–MIL–125,(nitro–explosives: TNP, TNT, RDX and o–DNB).

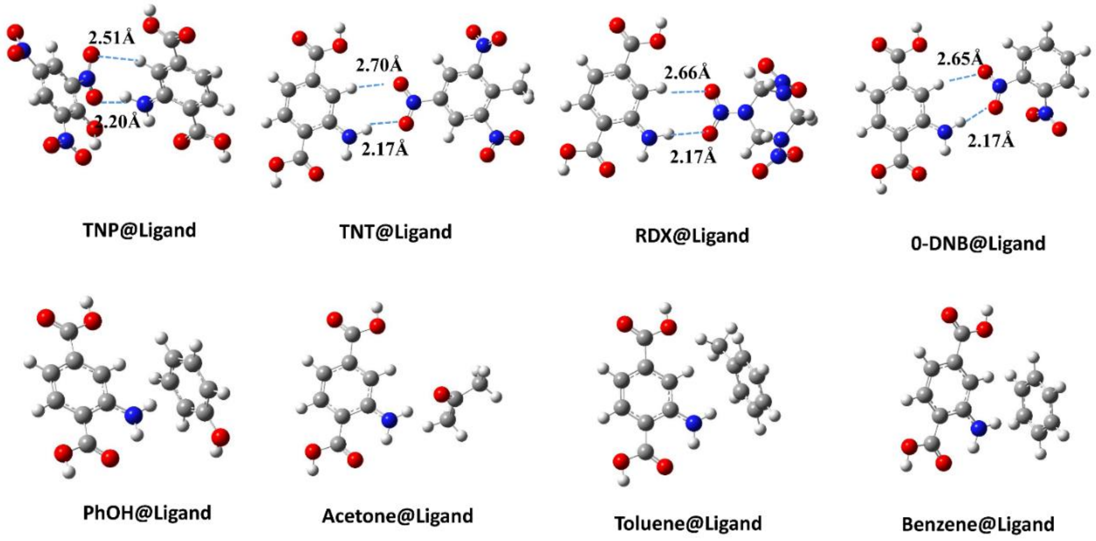

Fig. S24. Optimized structures of the studied conformers with different nitro–explosive binding to ligand.

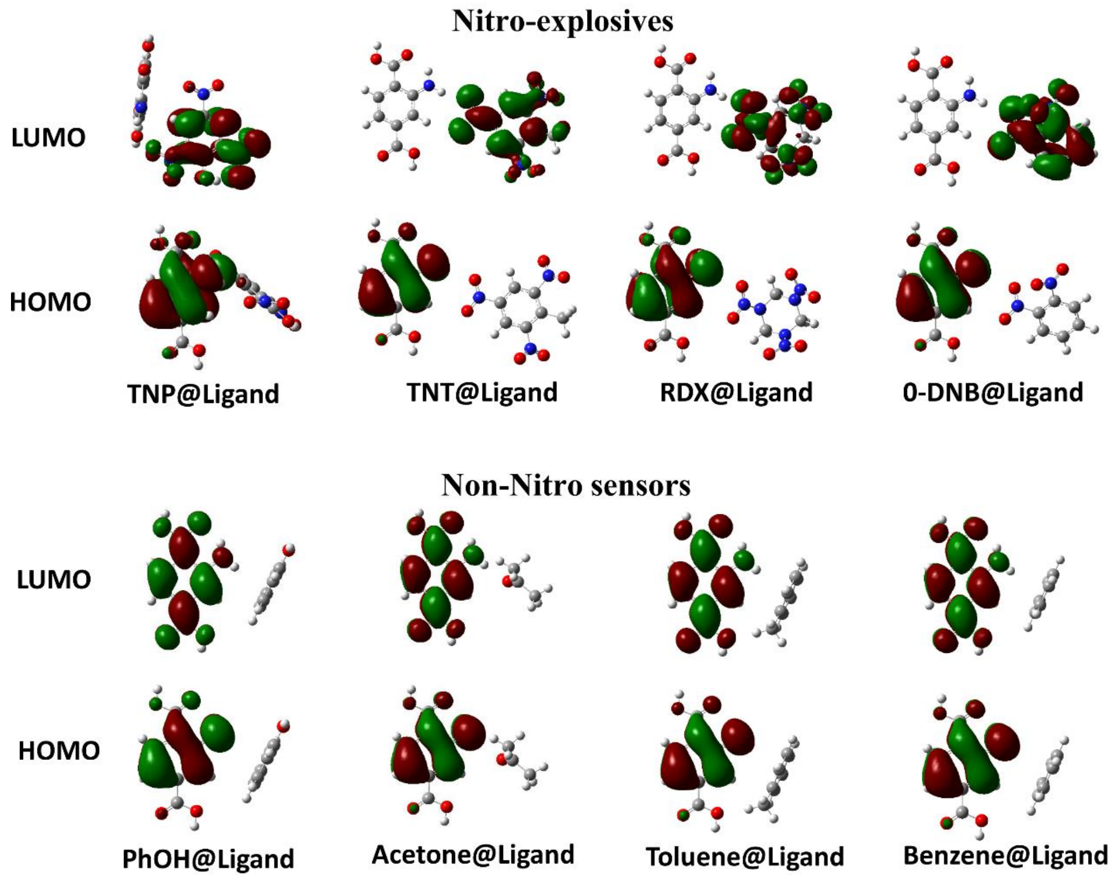

Fig. S25. HOMO and LUMO orbitals of the studied conformers.

Table S5. The HOMO and LUMO energies, HOMO–LUMO energy gaps, and the binding energy for the studied conformers.

	TiO_2	Metal node	Ligand	TNP@Ligand	TNT@Ligand	RDX@Ligand	o–DNB@Ligand	PhOH@Ligand	Acetone@Ligand	Toluene@Ligand	Benzene@Ligand
HOMO(eV)	–7.89	–8.12	–5.91	–6.04	–5.5	–5.49	–5.44	–5.71	–5.57	–5.74	–5.73
LUMO(eV)	–4.23	–3.30	–1.95	–3.94	–3.98	–2.65	–3.57	–1.87	–1.74	–1.91	–1.88
GAP(eV)	3.66	4.82	3.96	2.1	1.52	2.84	1.87	3.85	3.83	3.83	3.85
Binding energy (Kcal/mol)	–	–	–	–7.91	–8.11	–8.09	–8.73	–9.99	–10.35	–5.39	–6.25

Table S6. The calculated charge transfer integral (J_{eff}) of the charge transfer compounds of nitro–explosive binding to ligand.

	TNP@Ligand	TNT@Ligand	RDX@Ligand	o–DNB@Ligand
J_{eff} (meV)	32.9	18.5	10.01	13.2

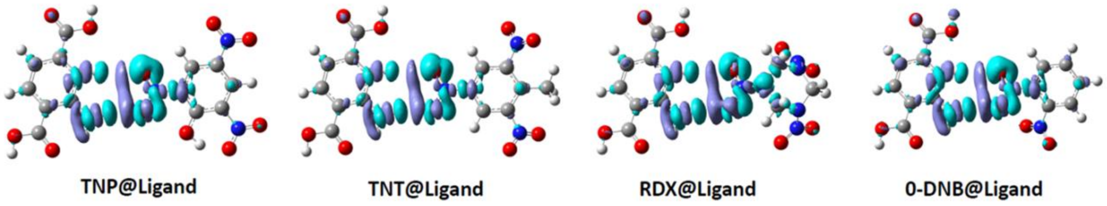

Fig. S26: Charge density difference of various nitro–explosives adsorbed on ligand, purple color denotes charge donor and cyan color denotes charge accept.

4. The mass–transduced microcantilever

We used a commercial microcantilever (RMC–100, High–End MEMS Technology, *Co. Ltd*., China) to evaluate the ability of NH_2–MIL–125 in concentrating nitro–explosive vapor. (**Fig. S27**). The specific gas molecule adsorption–induced mass–increase will proportionately cause a shift of the resonance frequency of the microcantilever. As can be seen in **Fig. S28,** the real–time adsorption curve of NH_2–MIL–125 to 4.9 ppt RDX and 9.1 ppb TNT at room temperature were recorded, respectively. The frequency shifts were observed upon the introduction of the target vapor. The pristine NH_2–MIL–125 coated on the monocrystal Si wafer exhibited frequency–shift responses of 55 Hz to 4.9 ppt RDX, and 48 Hz to 9.1 ppb TNT. The resulted frequency–shift can be re–transduced to the adsorption mass of explosive vapors by 1.05 Hz pg^{-1} ± 10%. Accordingly, the adsorption capacity of NH_2–MIL–125 to RDX and TNT gas were estimated to be 3.56×10^{-2} g cm^{-3} (± 10%) and 3.15×10^{-2} g cm^{-3} (± 10%), respectively. According to the equation of $PV = nRT$ and the estimated BET surface area of NH_2–MIL–125 (**Fig. S29**), the concentration of RDX and TNT in NH_2–MIL–125 materials were 3.92×10^{6} ppm (± 10%) and 3.39×10^{6} ppm (± 10%), respectively, which revealed the superior pre–concentration efficiency of RDX over TNT, and thus reduces the concentration differences of RDX and TNT at the interface of MOF and TiO_2.

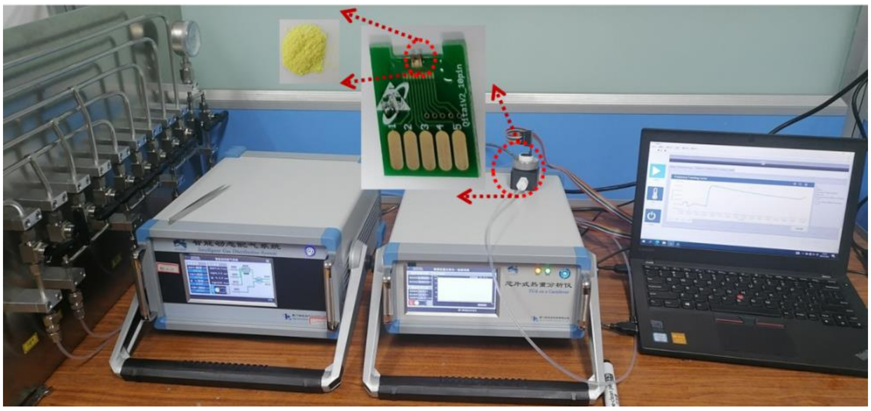

Fig. S27. Optical photographs of NH_2–MIL–125 loaded resonant microcantilever, where NH_2–MIL–125 (1873 pg) were loaded onto the free end of the microcantilever.

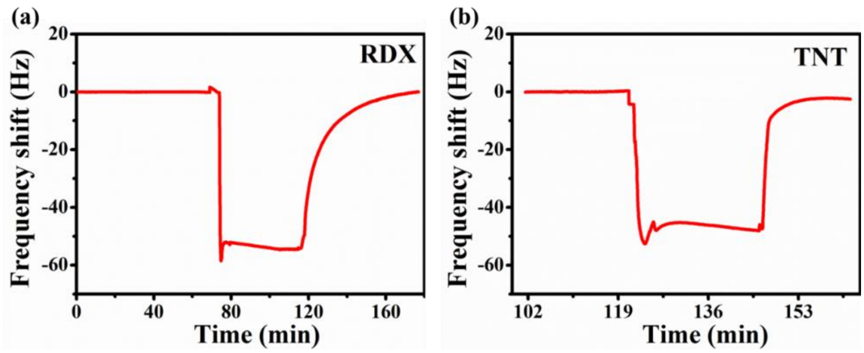

Fig. S28. Resonant–gravimetric sensing curves of NH_2–MIL–125 to (a) 4.9 ppt RDX, (b) 9.1 ppb TNT under dark. (noted: the recovery of resonance frequency to the baseline by in–situ heating at 35℃)

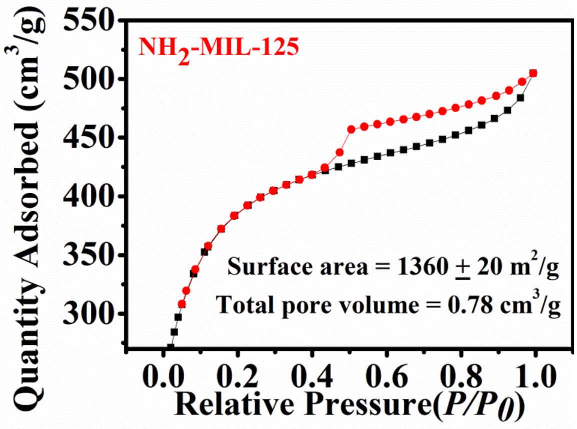

Fig. S29. N_2 adsorption–desorption isotherms for NH_2–MIL–125 at 77 K. Surface area and total pore volume of NH_2–MIL–125 are 1360 ± 20 and 0.78 cm^3/g, respectively.

$C_3H_6N_6O_6 + O_2^- \rightarrow [C_3H_6N_6O_6\text{-}O_2]^-$ **Eq. 1**

$C_3H_6N_6O_6 + 4O_2^- \rightarrow 4NO_2^- + 3CO_2 + 2NH_3$ **Eq. 2**

References

41. B. Liu, E. S. Aydil, Growth of Oriented Single–Crystalline Rutile TiO_2 Nanorods on Transparent Conducting Substrates for Dye–Sensitized Solar Cells. *J. Am. Chem. Soc.* **131**, 3985–3990 (2009).
42. R. F. Jin, Y. F. Chang, A theoretical study on photophysical properties of triphenylamine–cored molecules with naphthalimide arms and different pi–conjugated bridges as organic solar cell materials. *PCCP* **17**, 2094–2103 (2015).
43. G. M. Day *et al.*, A third blind test of crystal structure prediction. *Acta Crystallogr. Sect. B: Struct. Sci.* **61**, 511–527 (2005).
44. M. J. T. Frisch, G. W.;, H. B. Schlegel, Gaussian 09, Revision A.01. *Gaussian, Inc., Wallingford CT* (2009).
45. E. Aprà *et al.*, NWChem: Past, present, and future. *J. Chem. Phys.* **152**, 184102 (2020).
46. Z. Yang *et al.*, A High–Performance Nitro–Explosives Schottky Sensor Boosted by Interface Modulation. *Adv. Funct. Mater.* **25**, 4039–4048 (2015).
47. S. Jungsuttiwong *et al.*, Theoretical study on novel double donor–based dyes used in high efficient dye–sensitized solar cells: The application of TDDFT study to the electron injection process. *Org. Electron.* **14**, 711–722 (2013).
48. M. Schrader *et al.*, Comparative Study of Microscopic Charge Dynamics in Crystalline Acceptor–Substituted Oligothiophenes. *J. Am. Chem. Soc.* **134**, 6052–6056 (2012).
49. V. Ruehle *et al.*, Microscopic Simulations of Charge Transport in Disordered Organic Semiconductors. *J. Chem. Theory Comput.* **7**, 3335–3345 (2011).
50. J. Kirkpatrick, V. Marcon, K. Kremer, J. Nelson, D. Andrienko, Columnar mesophases of hexabenzocoronene derivatives. II. Charge carrier mobility. *J. Chem. Phys.* **129**, (2008).
51. M. D. Newton, Quantum chemical probes of electron–transfer kinetics – the nature of donor–acceptor interactions. *Chem. Rev.* **91**, 767–792 (1991).
52. K. Senthilkumar, F. C. Grozema, F. M. Bickelhaupt, L. D. A. Siebbeles, Charge transport in columnar stacked triphenylenes: Effects of conformational fluctuations on charge transfer integrals and site energies. *J. Chem. Phys.* **119**, 9809–9817 (2003).
53. K. Navamani, P. K. Samanta, S. K. Pati, Theoretical modeling of charge transport in triphenylamine–benzimidazole based organic solids for their application as host–materials in phosphorescent OLEDs. *RSC Adv.* **8**, 30021–30039 (2018).
